# Establishment of a new prognostic risk model of MAPK pathway-related molecules in kidney renal clear cell carcinoma based on genomes and transcriptomes analysis

**DOI:** 10.3389/fonc.2023.1077309

**Published:** 2023-03-10

**Authors:** Peizhi Zhang, Jiayi Li, Zicheng Wang, Leizuo Zhao, Jiechuan Qiu, Yingkun Xu, Guangzhen Wu, Qinghua Xia

**Affiliations:** ^1^ Department of Urology, Shandong Provincial Hospital, Cheeloo College of Medicine, Shandong University, Jinan, China; ^2^ School of Business, Hanyang University, Seoul, Republic of Korea; ^3^ Department of Urology, Shandong Provincial Hospital Affiliated to Shandong First Medical University, Jinan, China; ^4^ Department of Urology, Dongying People’s Hospital, Dongying, China; ^5^ Department of Breast and Thyroid Surgery, The First Affiliated Hospital of Chongqing Medical University, Chongqing, China; ^6^ Department of Urology, The First Affiliated Hospital of Dalian Medical University, Dalian, China

**Keywords:** KIRC, tumor biomarkers, MAPK pathway, TCGA, prognostic model

## Abstract

**Purpose:**

The mitogen-activated protein kinase (MAPK) signaling pathway is often studied in oncology as the most easily mentioned signaling pathway. This study aims to establish a new prognostic risk model of MAPK pathway related molecules in kidney renal clear cell carcinoma (KIRC) based on genome and transcriptome analysis.

**Methods:**

In our study, RNA-seq data were acquired from the KIRC dataset of The Cancer Genome Atlas (TCGA) database. MAPK signaling pathway-related genes were obtained from the gene enrichment analysis (GSEA) database. We used “glmnet” and the “survival” extension package for LASSO (Least absolute shrinkage and selection operator) regression curve analysis and constructed a prognosis-related risk model. The survival curve and the COX regression analysis were used the “survival” expansion packages. The ROC curve was plotted using the “survival ROC” extension package. We then used the “rms” expansion package to construct a nomogram plot. We performed a pan-cancer analysis of CNV (copy number variation), SNV (single nucleotide variant), drug sensitivity, immune infiltration, and overall survival (OS) of 14 MAPK signaling pathway-related genes using several analysis websites, such as GEPIA website and TIMER database. Besides, the immunohistochemistry and pathway enrichment analysis used The Human Protein Atlas (THPA) database and the GSEA method. Finally, the mRNA expression of risk model genes in clinical renal cancer tissues versus adjacent normal tissues was further verified by real-time quantitative reverse transcription (qRT-PCR).

**Results:**

We performed Lasso regression analysis using 14 genes and created a new KIRC prognosis-related risk model. High-risk scores suggested that KIRC patients with lower-risk scores had a significantly worse prognosis. Based on the multivariate Cox analysis, we found that the risk score of this model could serve as an independent risk factor for KIRC patients. In addition, we used the THPA database to verify the differential expression of proteins between normal kidney tissues and KIRC tumor tissues. Finally, the results of qRT-PCR experiments suggested large differences in the mRNA expression of risk model genes.

**Conclusions:**

This study constructs a KIRC prognosis prediction model involving 14 MAPK signaling pathway-related genes, which is essential for exploring potential biomarkers for KIRC diagnosis.

## 1 Introduction

The mortality rate of kidney cancer ranks first among all urological malignancies (1). Renal cell carcinoma (RCC) is the most common type of primary renal malignancy, and about 70% of RCC patients are diagnosed with KIRC (2). More than one-fifth of patients with advanced kidney cancer will relapse even after radical nephrectomy. Besides, kidney cancer patients with distant metastases have a 1-year survival rate of only 50% and a 5-year survival rate of only 10% (3, 4). Early diagnosis and treatment are of great importance to improve the prognosis of kidney cancer. A growing number of studies confirm that cancer is a human genomic disease (5, 6). Tumor progression is caused by coordinated genetic changes in multiple signaling pathways (7). Therefore, it is important to explore the relevant cancer-causing genes and pathways and construct risk models based on them for early detection and treatment of KIRC.

MAPK (mitogen-activated protein kinase) signaling pathway is one of the most extensive pathways in tumor pathway research. Related studies in human cancers have confirmed that most of cancers are associated with changes in the MAPK pathway. Since the recognition of Ras small GTPases as the first oncogenes of sarcoma viruses, research on the MAPK pathway has intensified over the past 40 years (8). The internal signaling of the MAPK signaling pathway is complex. Besides, this signaling pathway is often regulated by related genes or by crosstalk with other signaling pathways. In the physiological state, intracellular MAPK signaling is tightly controlled. Growth factors (GFs) bind to and activate receptor tyrosine kinases (RTKs) on the cell membrane, a critical first step in initiating the classical MAPK signaling pathway (9). Activation of RTKs drives phosphorylation of RAS superfamily proteins represented by HRAS, KRAS, and NRAS, thereby transducing extracellular signals to the cytoplasm (10). The subsequent activation of intracellular cascade reactions is also caused by the phosphorylation of molecules. Activated RAS further activates MAPKKK (mitogen-activated protein kinase kinase, represented by RAF and its variants), followed by MAPK kinase (MAPKK: MEK1/2/3/4/5/6/7), and finally MAPK, resulting in a cascade activation reaction of the intracellular MAPK signaling pathway (11). The MAPKs mainly include the following: ERKs(extracellular signal-regulated kinases, represented by ERK1/2/5), JNKs(c-Jun N-terminal kinases, represented by JNK1/2/3), and p38 MAPKs(represented by p38α/β/γ/δ) (12–14). Numerous studies have confirmed that the progression of most solid tumors is associated with gene mutations in the RAS/RAF/MEK/ERK signaling pathway (15). Approximately 30% of human solid tumors are associated with mutations in the RAS gene (16). Activation of Ras not only drives the MAPK cascade, but also acts as an initiator of the PI3k/AKT/mTOR cascade to regulate cell growth (11). In addition, ERK1/2 can regulate the activation of transcriptional factors such as c-Myc (transcriptional regulator Myc-like) through phosphorylation, which has received much attention in the research of tumor-targeted therapy (12).

In recent years, studies have demonstrated that the MAPK signaling pathway influences the prognosis of KIRC through the regulation of HIF-1α (17). In addition, the MAPK signaling pathway also influences the sensitivity of KIRC patients to targeted drugs such as sunitinib and sorafenib (17, 18). The construction of predictive models based on genes related to the MAPK signaling pathway and the exploration of the mechanisms by which the MAPK signaling pathway affects prognosis and targeted therapy resistance will be of great significance in the future for the diagnosis and treatment of KIRC.

## 2 Materials and methods

### 2.1 Data acquisition

The mRNA expression data and clinical datasets of KIRC patients used in this study were obtained from the TCGA database (https://portal.gdc.cancer.gov/). The dataset we downloaded included 539 tumor tissues and 72 normal tissues. We then downloaded and analyzed the MAPK pathway-related genes using the GSEA analysis website (https://www.gsea-msigdb.org/gsea/index.jsp).

### 2.2 Data processing and analysis

The R language operating platform (https://www.rstudio.com/) is one of the most influential and widely used bioinformatics operating platforms. We used Perl and several R packages to analyze and process data. The “heatmaps” expansion package was used to make the heatmap. Then we used tbtools (https://github.com/CJ-Chen/TBtools) to further beautify and process the heatmap to better display the data. Statistical data analysis was performed using the “limma” software package to analyze variance. Lasso regression analysis was mainly performed using “glmnet” expansion packages. The survival curve was plotted using the “survival” expansion packages, and the ROC curve was analyzed and plotted using the “survival ROC” extension package. Finally, based on the risk model, we validated it with clinical characteristics by univariate Cox analysis and multivariate Cox analysis using the “survival” and “forestplot” expansion packages. Finally, we combined the predictive risk model with various clinical features as independent risk factors to draw a nomogram using the “rms” expansion package. P <0.05 was considered a statistically significant difference. We used the “plyr”, “ggplot2”, “grid” and “gridExtra” extension packages for multi-GSEA analysis, to explore the biological pathways that risk model genes may affect in KIRC, and to explore the correlation of the MAPK pathway with other pathways.

### 2.3 GEPIA website

GEPIA (http://gepia.cancer-pku.cn/) has a robust data aggregation function. The analysis tool includes RNA-seq expression data from more than 9,000 tumors and 8,000 tumor genome maps based on the TCGA database (19). Based on the website’s online tool, the CNV and SNV of model genes were differentially analyzed in different tumors.

### 2.4 ImmuCellAI website

We analyzed the infiltration of 24 types of immune cells in pan-cancer based on the ImmuCellAI website (http://bioinfo.life.hust.edu.cn/ImmuCel lAI/). We used the “pheatmap” R language to draw and visualize the analysis results in the form of heat maps. Statistical analysis was performed using the Spearman’s correlation coefficient.

### 2.5 Generation of PPI networks

We draw the PPI network based on the online analysis tool STRING (https://www.string-db.org/). To make the PPI network more beautiful, we used the visualization software of Cystoscope. The data in PPI were used to construct a quantization table.

### 2.6 TIMER website

The Tumor Immune Estimation Resource (TIMER) 2.0s (http://cistrome.org/TIMER/) has recently been used to analyze immune cell infiltration in the environment of tumors. This study further judged the infiltration of immune cells in 14 genes by analyzing the correlation between 14 genes and immune cells. Heatmaps were drawn and visualized using the “heatmaps” expansion package.

### 2.7 GDSC database

Two hundred sixty-six drugs are included in the GDSC database (20). In this study, we analyzed the relationship between related drugs and the mRNA expression of MAPK pathway-related genes based on the GDSC database, and then we drew a heatmap to visualize the correlation.

### 2.8 The Human Protein Atlas database

The Human Protein Atlas database (http://www.proteinatlas.org/) was a proteome analysis website of 27173 antibodies targeting 17268 unique proteins (21). In our study, we used this website to explore the protein expression of MAPK pathway-related genes in normal kidney tissues and ccRCC tumor tissues.

### 2.9 Collection of clinical tissue samples

From March to May 2022, we collected tumor and adjacent normal kidney samples of 8 KIRC patients from Shandong Provincial Hospital. This study was approved by the Ethics Committee of Shandong Affiliated Hospital. Patients provided written informed consent for all samples and information collected. The research adhered to the principles of the Declaration of Helsinki and those of the World Medical Association.

### 2.10 Total RNA extraction and qRT-PCR experiments

We extracted total RNA from collected KIRC tumor tissues and paracancerous normal tissues using TRIzol reagent (Thermo Fisher Scientific, Waltham, MA, USA). Next, we reverse-transcribed the pre-extracted RNA into cDNA using EvoM-MLVRT master mix (Accurate Biotechnology). We then mixed the reagents for qRT-PCR detection according to the manufacturer’s instructions of the SYBR^®^ Green Premix Pro Taq HS qPCR Kit (Accurate Biotechnology). The above process was carried out in strict accordance with the manufacturer’s instructions.

### 2.11 Statistical analyses

Expression of MAPK pathway-related genes in tumor tissues and adjacent tissues using One-way ANOVA. T-test was used to compare the expression of MAPK pathway-related genes of different gender, age, stage, node (N), tumor (T) and metastasis (M) in KIRC data set. The “survminer” package was used to determine the cut-off value of each risk score in the tumor group, and we divided patients into a high-risk group and a low-risk group. R Studio software package was used for all statistical analysis. P < 0.05 meant statistically significant.

## 3 Results

### 3.1 The expression of MAPK signaling pathway-related genes in KIRC and univariate Cox analysis

**Figure 1:**
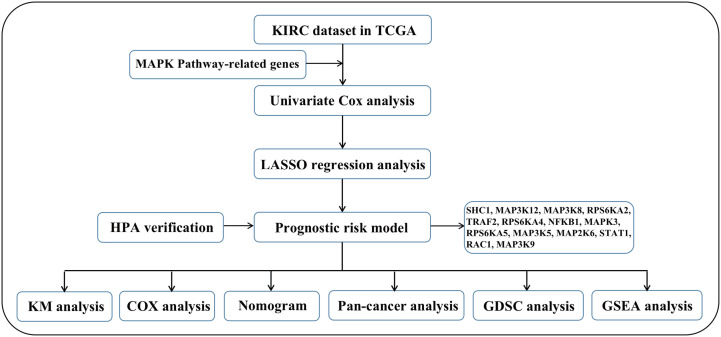
The flow chart of this research.

**Figure 2:**
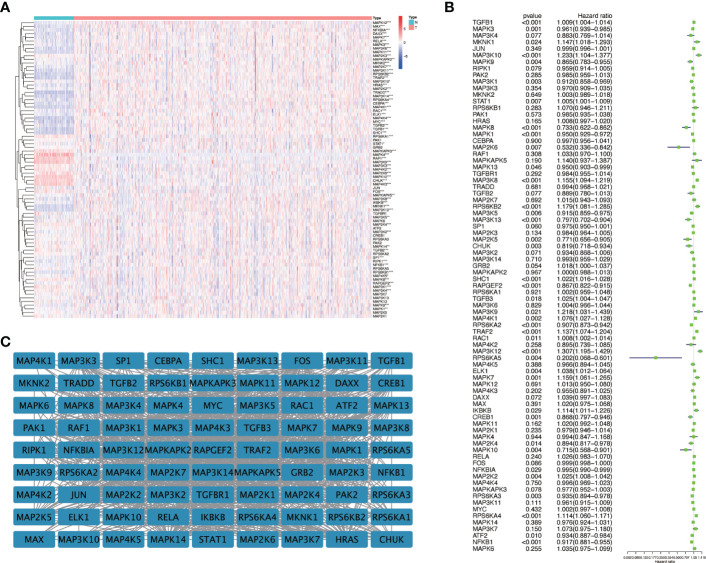
The expression of genes related to the MAPK signaling pathway in KIRC and univariate Cox regression analysis. **(A)** The differential expression of 81 MAPK signaling pathway-related genes in cancer and normal tissue in KIRC patients. Red represents the gene that is highly expressed in the tumor. The darker the color, the higher the expression level; blue represents the gene that is lowly expressed in the tumor, and the color the deeper it is, the lower the expression level. **(B)** Perform univariate Cox regression analysis on genes related to the MAPK signaling pathway. **(C)** The String database was used to analyze the protein interaction of 81 genes related to the MAPK signaling pathway, and the Cystoscope software platform was used to visualize the analysis results. *P<0.05, **P<0.01, and ***P<0.001.

We first drew the flowchart to more conveniently show this research process (**Figure 1**). Then, We constructed a heat map of the mRNA data of the 81 MAPK signaling pathway-related genes in the KIRC patient dataset based on the TCGA database (**Figure 2A**). Among the 81 MAPK signaling pathway-related genes, nearly 80% of the genes have statistically significant differences in expression between normal kidney tissue and KIRC tissue, further confirming that the MAPK signaling pathway plays an essential role in the occurrence and development of KIRC. We then performed the univariate Cox analysis of MAPK signaling pathway-related genes in KIRC patients, and drew a forest plot (**Figure 2B**). The potential role of each signaling pathway-related gene in the occurrence and development of KIRC was determined. Using the HR value of 1 as a cutoff, there are 16 genes with HR values >1, including STAT1(signal transducer and activator of transcription 1), MAP3K8(mitogen-activated protein kinase kinase 8), SHC1(SHC adaptor protein 1), MAP3K9(mitogen-activated protein kinase kinase kinase 9), TRAF2(TNF receptor associated factor 2), RAC1(Rac family small GTPase 1), MAP3K12(mitogen-activated protein kinase kinase kinase 12), RPS6KA4(ribosomal protein S6 kinase A4), meaning that these genes are risk factors in disease progression. whereas 23 genes, including MAPK3(MAPK3: mitogen-activated protein kinase 3), MAP2K6(MAP2K6: mitogen-activated protein kinase kinase 6), MAPK13, MAP3K5(mitogen-activated protein kinase kinase 5), RPS6KA2(ribosomal protein S6 kinase A2), RPS6KA5(ribosomal protein S6 kinase A5), NFKB1(nuclear factor kappa B subunit 1), whose HR values are less than 1, are protective factors. Finally, we used the String database to analyze the PPI protein interaction to verify the interaction and connection between the proteins in the MAPK pathway (**Figure 2C**).

### 3.2 Construct a novel prognostic-related survival model in KIRC

**Figure 3:**
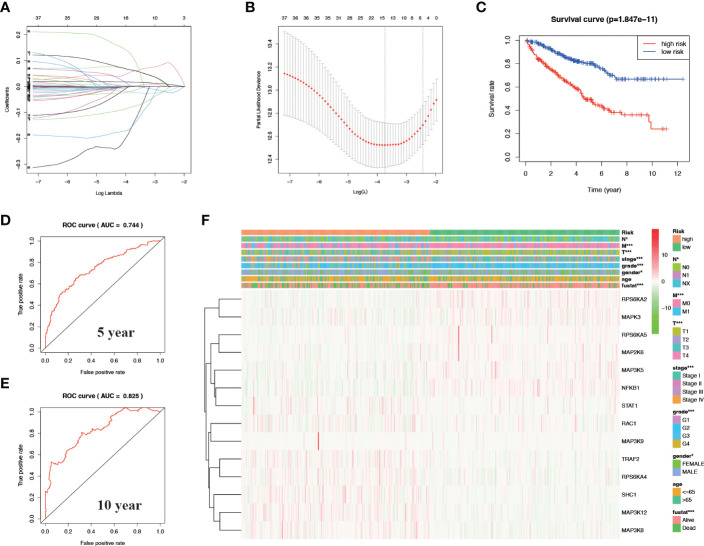
Construct a prognostic-related risk model in KIRC through LASSO regression analysis. **(A, B)** Results of LASSO regression analysis and cross-validation. **(C)** Kaplan–Meier survival analysis between high-risk and low-risk groups according to the optimal cut-off value; **(D)** ROC curve for predicting 5-year survival time; **(E)** ROC curve for predicting 10-year survival time; **(F)** Heat map based on the correlation of this risk feature with clinical features. *P<0.05 and ***P<0.001.

After univariate cox analysis of genes related to the MAPK signaling pathway, we screened out genes with a P value < 0.05 for LASSO regression analysis, and screened out 14 model genes, including RPS6KA2, MAPK3, RPS6KA5, MAP2K6, MAP3K5, NFKB1, STAT1, RAC1, MAP3K9, TRAF2, RPS6KA4, SHC1, MAP3K12, and MAP3K8 (**Figures 3A**, **B**). A prognostic risk model was established based on these model genes. KIRC patients were divided into high-risk and low-risk groups with the median level of risk score as the optimal cutoff value. After plotting the survival curves, we found a significant difference in survival between the two groups (**Figure 3C**). Subsequently, we validated this prognostic-related risk model using the ROC curve. The results showed that the 5-year AUC value was 0.744 (**Figure 3D**) and the 10-year AUC value was 0.825 (**Figure 3E**), suggesting that the risk model is suitable for prognosis prediction of KIRC patients with high accuracy.

### 3.3 The relationship between the risk model and clinicopathological characteristics, and draw the corresponding nomogram in KIRC

**Figure 4:**
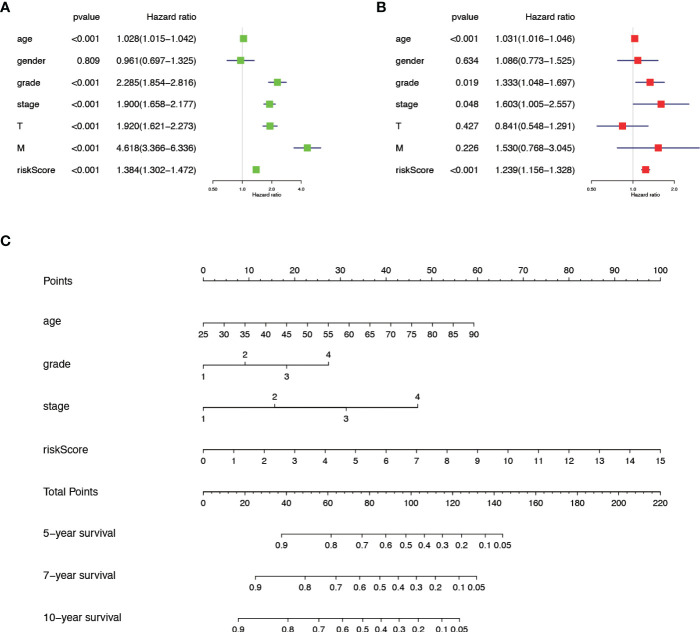
The comprehensive analysis is based on the clinical information of KIRC patients. **(A)** Univariate Cox analysis. **(B)** Multivariate Cox analysis. **(C)** A new nomogram was drawn based on this prognostic risk signature. The value of each variable gets a score on the dot scale axis. The total score can be easily calculated by adding each score and projecting the total score to a lower total score system. We can estimate the risk for predicting 5-, 7- or 10-year survival in KIRC.

We verified the relationship between the prognostic risk model and the clinical characteristics of patients (**Figure 3F**). The prognostic risk model was correlated with clinical characteristics including tumor volume (T), lymph nodes (N) distant metastasis (M), stage, grade, gender, and fustat, suggesting that the predictive model has good clinical prognosis and diagnostic and therapeutic efficacy. Univariate Cox analysis found that age, stage, grade tumor volume (T), distant metastasis (M), and risk score were statistically significant (**Figure 4A**). Multivariate Cox analysis showed that age, stage, grade, and risk score were independent risk factors for KIRC (**Figure 4B**). Subsequently, we established a new nomogram based on the four independent risk factors verified by multivariate Cox analysis (**Figure 4C**). In this nomogram, the quantified values of each variable correspond to the scale axis to obtain a score. Finally, the total score is obtained by summing the scores corresponding to the four variables, so that the 5-, 7-, or 10-year survival of KIRC patients can be intuitively obtained.

### 3.4 OS and variation of model genes in pan-cancer

**Figure 5:**
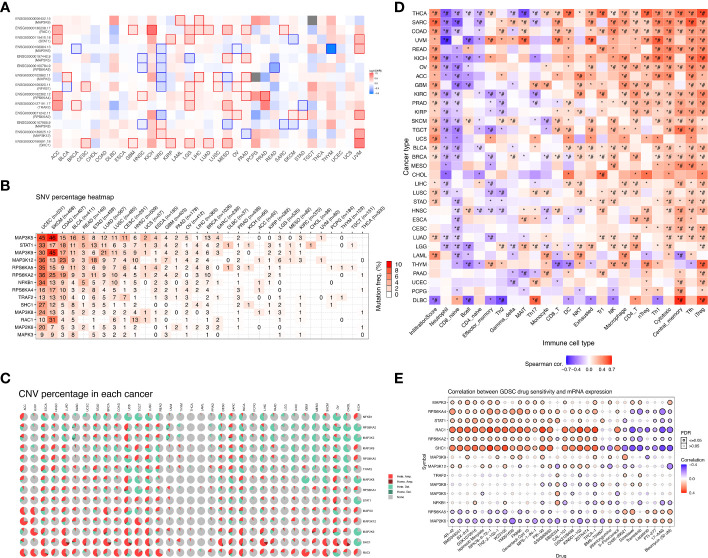
Overall survival analysis and variation analysis of this risk model gene in pan-cancer. **(A)** Overall survival analysis of this risk model gene in pan-cancer. Red represents this risk model gene as a risk factor, and blue represents this risk model gene as a protective factor. **(B)** SNV levels of 14 model genes in pan-cancer, where the darker the red color, the higher the probability of SNV. **(C)** CNV ratio of 14 model genes in pan-cancer, Light red hete amp represents heterozygous amplification, light green hete del represents heterozygous deletion, dark red Homo amp represents homozygous amplification, dark green Homo del represents homozygous deletion, and gray represents no CNV. **(D)** The GSVA method was used to analyze the level of immune cell infiltration in 33 different types of tumors, and the Spearman correlation coefficient was used to evaluate its correlation. Red indicates that the level of immune cell infiltration is positively correlated with the tumor. On the contrary, blue indicates a negative correlation. (^*^P-value ≤ 0.05; ^#^FDR ≤ 0.05). **(E)** In a sensitivity analysis of prognostic risk model gene mRNA expression and mainstream anticancer drugs, red represents a positive correlation, while blue represents a negative correlation.

We mapped the mRNA expression, CNV and SNV of these genes in 33 different tumors. First, we observed the extent to which these 14 model genes affect survival and prognosis in pan-cancer (**Figure 5A**). When we explored the role of genes in different tumors, we found that genes such as RAC1 and SHC1 were elevated in most cancers, suggesting their role as prognostic risk factors in most tumors. For a specific tumor pathological type, we can observe that most MAPK signaling pathway model genes are highly expressed in KICH and LGG, suggesting that they are associated with poor prognosis. Notably, we found that high expression of MAP2K6, MAP3K5, RPS6KA5, MAPK3, NFKB1, and RPS6KA4 in KIRC tumors suggested a better prognosis. In contrast, high expression of MAP3K8 and MAP3K12 suggested a poorer prognosis for KIRC. The SNV percentage heatmap (**Figure 5B**) and CNV percentage (**Figure 5C**) heatmap show the single nucleotide variation and copy number variation of different model genes in pan-cancer, respectively. The SNV percentage heatmap found that MAP3K5, STAT1, and MAP3K9 have the highest single-nucleotide mutation rates in pan-cancer. When we explored the single-nucleotide mutations of pathway-related genes in various pathological types of tumors, we found that the MAPK signaling pathway prognostic model genes had the most obvious SNV in uterine corpus endometrial carcinoma (UCEC), skin cutaneous melanoma (SKCM), and colon adenocarcinoma (COAD). In particular, the single-nucleotide mutation rate of MAP3K5 in UCEC and SKCM tumors was as high as 45% and 46%, respectively, while the single-nucleotide mutation rate of MAP3K9 in SKCM tumors was 46%. Nucleotide mutations played an essential role in the development of these tumors. Next, we found copy number variations of MAP2K6, SHC1, and RAC1 in most cancer tissues. RPS6KA2, MAP3K5, MAP3K9, RPS6KA5, and TRAF2 had higher rates of heterozygous deletion mutations in KIRC tissue, while STAT1, MAPK3, MAP3K12, SHC1, and RAC1 heterozygous amplification mutations were more prevalent. Notably, the MAPK pathway model genes had a significantly increased mutation rate in KICH, which was one of the most common pathological types of RCC.

### 3.5 Immune infiltration and drug sensitivity of model genes in pan-cancer

We verified the correlation of the risk model genes with the infiltration of various immune cells in different types of tumors (**Figure 5D**). DC, NKT, Tr1, NK, Macrophage, CD4_T, nTreg, Th1,Tfh, and iTreg show high expression in most types of tumors, suggesting that their infiltration potentially contributes to tumor progression. On the contrary, Neutrophil and CD8_naive were lowly expressed in most types of tumors. Notably, immune cells such as NKT, Tr1, NK, macrophages, CD4_T, nTreg, Th1, Tfh, and iTreg were more infiltrated in KIRC tissues, while neutrophils, CD8_naive, CD4_naive, Th2, and Th17 were less infiltrated. Based on the establishment of the previous prediction model, we analyzed the correlation between the mRNA expression of 14 model genes and drug sensitivity (**Figure 5E**). Drug sensitivity analysis showed that MAPK3, RPS6KA4, STAT1, RAC1, RPS6KA2, SHC1 and other model genes, especially RAC1 and SHC1 genes, were significantly positively correlated with drug sensitivity. On the contrary, the higher the expression of RPS6KA5, MAP2K6 and other genes, the worse the drug sensitivity and the worse the curative effect.

### 3.6 Verify the protein expression of model genes between KIRC tissues and normal tissues

**Figure 6:**
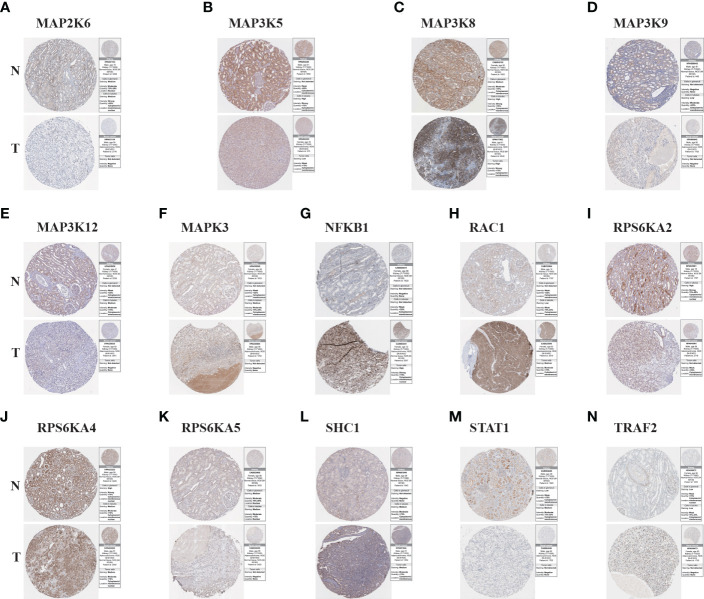
The Results of immunohistochemistry. **(A–N)** The Human Protein Atlas database was used to verify the proteins’ differential expression of 14 model genes (MAP2K6, MAP3K5, MAP3K8, MAP3K9, MAP3K12, MAPK3, NFKB1, RAC1, RPS6KA2, RPS6KA4, RPS6KA5, SHC1, STAT1, TRAF2) in KIRC tumor tissues (T) and adjacent normal tissues (N).

To further understand the protein expression of 14 model genes in KIRC tumor and normal tissues, we used the HPA website for further analysis (**Figures 6A–N**). We found that MAP2K6, MAP3K5, MAP3K9, MAP3K12, RPS6KA2, RPS6KA5, and STAT1 were lowly expressed in tumor tissues; However, NFKB1, RAC1, SHC1, and TRAF2 are highly expressed compared to normal tissues. The above results are consistent with our previous verification results.

### 3.7 GSEA analysis in KIRC for risk model genes

**Figure 7:**
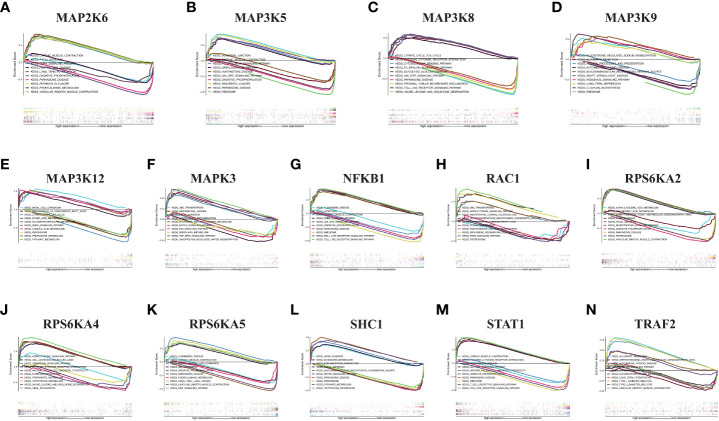
GSEA in KIRC. **(A)** MAP2K6. **(B)** MAP3K5. **(C)** MAP3K8. **(D)** MAP3K9. **(E)** MAP3K12. **(F)** MAPK3. **(G)** NFKB1. **(H)** RAC1. **(I)** RPS6KA2. **(J)** RPS6KA4. **(K)** RPS6KA5. **(L)** SHC1. **(M)** STAT1. **(N)** TRAF2.

We performed GSEA pathway analysis on these risk model genes to explore the role of MAPK-related genes in other pathways and to establish the connection between the MAPK pathway and other pathways (**Figures 7A–N**). We found that risk model genes play different roles in different pathways, and each gene is also involved in different signaling pathways. For example, MAP2K6 is elevated in focal adhesion, adhesion, long-term potentiation, vascular smooth muscle contraction, GnRH signaling pathway, pathways in cancer, but its expression decreased in Parkinson disease, oxidative phosphorylation, phenylalanine metabolism.

### 3.8 Validation of mRNA differential expression of risk model genes in KIRC clinical samples based on qRT-PCR

**Figure 8:**
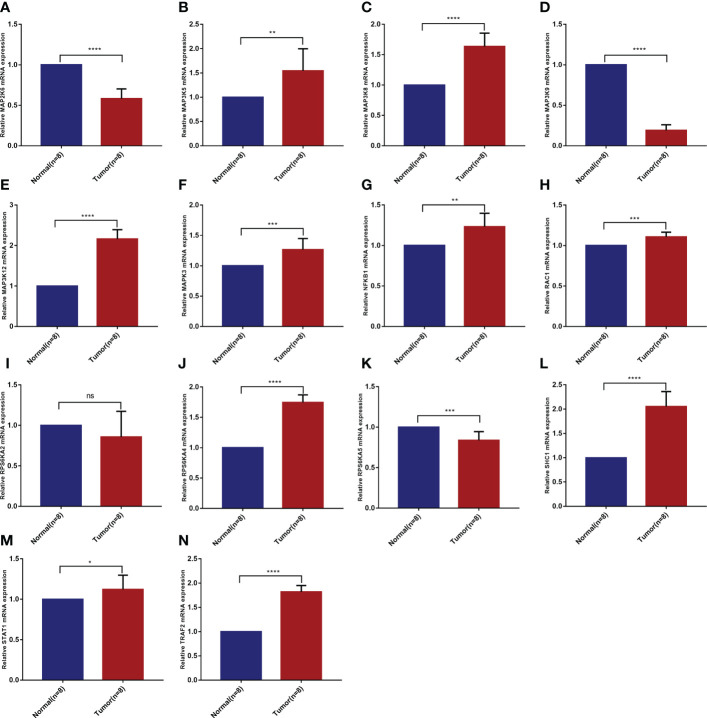
Validation of mRNA differential expression of risk model genes between KIRC pathological tissues and normal control tissues based on qRT-PCR. **(A)** MAP2K6. **(B)** MAP3K5. **(C)** MAP3K8. **(D)** MAP3K9. **(E)** MAP3K12. **(F)** MAPK3. **(G)** NFKB1. **(H)** RAC1. **(I)** RPS6KA2. **(J)** RPS6KA4. **(K)** RPS6KA5. **(L)** SHC1. **(M)** STAT1. **(N)** TRAF2. *p < 0.05, **p < 0.01, ***p < 0.001, ****p < 0.0001, ns means no significance.

Based on the analysis of public databases, we successfully screened out 14 risk model genes. To further verify the reliability of the previous experimental results and evaluate the clinical application value, we collected 8 pairs of KIRC pathological tissues and normal control tissues. Based on qRT-PCR experiments, we verified the samples’ relative mRNA expression levels of 14 risk model genes (**Figures 8A–N**). We found that most genes (including MAP3K5, MAP3K8, MAP3K12, MAPK3, NFKB1, RAC1, RPS6KA4, SHC1, STAT1 and TRAF2) were increased in KIRC pathological tissues. In contrast, the mRNA expression levels of MAP2K6, MAP3K9 and RPS6KA5 in KIRC pathological tissues were reduced to varying degrees compared with normal control tissues. The mRNA expression of RPS6KA2 was not statistically significant in the difference analysis.

## 4 Discussion

In 2020, experts estimated 431,288 new kidney tumors worldwide, and 179,368 patients worldwide died from kidney cancer in the same year (22). Renal cell carcinoma (RCC) originates from renal cortical or tubular epithelial cells, of which KIRC is the most common subtype. The current treatment methods for early KIRC are mainly limited to surgery, and patients often have a good prognosis after surgery (23). However, although the targeted therapies has brought the light of treatment to advanced stage KIRC patients who are ineligible for surgery, drug resistance and side effects have resulted in a median survival of less than 3 years (24). Precision medicine has always been the development trend of current medical diagnosis and treatment, and the establishment of new predictive models will have a positive effect on the early diagnosis of cancers. To this end, we comprehensively used bioinformatics analysis tools and websites to analyze MAPK pathway-related genes in pan-cancer and establish a predictive model in KIRC. In addition, we validated these prognostic genes in KIRC tissues. We hope that this study will provide guidance for the early diagnosis and targeted treatment of KIRC.

We used 14 risk model genes in pan-cancer for CNV, SNV, drug sensitivity, immune infiltration, and overall survival analysis, and predicted other biological pathways that these 14 MAPK pathway-related genes may be involved in. Since the main area of focus of this study is KIRC, we discuss KIRC in more depth. Our study first analyzed the mRNA expression of 81 MAPK pathway-related genes in KIRC patients and normal kidney tissues. The results indicated that nearly 80% of the genes were differentially expressed. Research statistics show that over 85% of cancers have overactive MAPK signaling, which is directly caused by genetic changes in its upstream activators or key molecules (including RTK, RAS, and BRAF) or affected by changes in other regulatory genes (25). These results also demonstrate that altered expression of MAPK pathway-related genes may influence KIRC progression by affecting MAPK signaling pathway transduction. Precision medicine has always been the development trend of current medical diagnosis and treatment, and the establishment of new predictive models has led the way in the diagnosis and treatment of cancers. After univariate COX and LASSO regression analysis, we established a risk model consisting of 14 MAPK pathway-related genes, including RAC1, SHC1, NFKB1, MAPK3, RPS6KA2, RPS6KA4, RPS6KA5, MAP3K5, MAP3K8, MAP3K9, MAP3K12, STAT1, TRAF2, MAP2K6.

RAC1 belongs to the RAS superfamily of small GTP-binding proteins. This molecule often acts as an upstream of the MAPK signaling pathway and is often used as a target for tumor therapy (26). RAC1 inhibitors, such as the compound GYS32661 proved to be effective in tumor therapy. Our investigation further confirmed that RAC1 is highly expressed in ccRCC at the mRNA and protein levels. Further investigation of RAC1 may provide a basis for the therapeutic application of RAC1 inhibitors in ccRCC. The role of SHC1 in the MAPK signaling pathway is mainly to link activated receptor tyrosine kinases to the Ras, which in turn participates in the MAPK signaling cascade. Recent studies have confirmed that SHC1 interacts to form protein complexes to promote the progression of lung cancer (27). This is consistent with the trend of elevated expression of SHC1 in ccRCC in our study. NFKB1, a common transcription regulator, acts as a transcriptional regulator and contributes to the infiltration of inflammatory cells by moving to the nucleus when it is activated. The present study demonstrated that NFKB1 mRNA was highly expressed in ccRCC. A related study confirmed that the expression of HIF-1α decreased dramatically in ccRCC cells due to the reduced movement of NF-kB1 to the nucleus, which also inhibited the progression of ccRCC (28). The above results also confirm that the decreased expression of NFKB1 in ccRCC may be associated with the inhibition of tumor progression. MAPK3 encodes a protein that is an important member of the MAP kinase family. MAPK3/ERK1 plays a critical role in the MAPK/ERK cascade. As a recognized oncogene, its role in promoting cancer progression and influencing drug resistance to targeted drugs has been demonstrated in a variety of cancers (29, 30). Mutations in BRCA1-associated protein-1 (BAP1) are very common in ccRCC, and Jin S et al. used PPI network analysis to confirm that mutations in MAPK3, one of the core genes, regulated BAP1 (31). Our study also confirmed the increased mRNA expression of MAPK3 in ccRCC, and whether it could regulate BAP1 to affect the prognosis of ccRCC needs to be further investigated. RPS6KA2, RPS6KA4, and RPS6KA5 belong to the RSK (ribosomal S6 kinase) family of serine/threonine kinases. The common characteristics of this family are that they all have kinase catalytic domains, which can phosphorylate various MAPK signaling pathway-related molecules. Milosevic N et al. showed that RPS6KA2 acts downstream of EGFR/RAS/MEK/ERK signaling and is activated by EGF. Inhibition of its activity could synergize with erlotinib against pancreatic cancer cell survival (32). RPS6KA5 regulates lung tumor growth by activating the MAPK classical signaling pathway through phosphorylation, which in turn phosphorylates TRIM7 protein (33). RPS6KA4 is activated by the RAS-MAPK or p38-MAPK pathway and activates histone H3 by phosphorylation, leading to increased transcription of genes such as proto-oncogene c-fos/FOS and c-jun/JUN (34). MAP3K5, MAP3K8, MAP3K9, and MAP3K8 all belong to the serine/threonine protein kinase family. The above four kinases have been extensively studied in different types. MAP3K8 is a common oncogene in most tumors. Our study likewise confirmed the high expression of MAP3K8 in ccRCC. This molecule can mediate the MAPK signaling pathway by activating MAP kinase and JNK kinase pathways. Many studies have shown that some striking features of the tumor microenvironment can promote immunosuppression and limit the anticancer immune response. Among them, immune cells infiltrating the physical barrier and causing local inflammation play an essential role in forming and developing tumors (35). MAP3K8 also promotes the production of TNF-alpha and IL-2 during T-lymphocyte activation, which also links the MAPK signaling pathway to immune cell infiltration (36–38). STAT1 can be activated by EGF phosphorylation, thus forming a dimer that is transferred to the nucleus to act as a transcriptional activator. Most evidence suggests that STAT1 plays an oncogenic role in tumor cells. However, results from several experimental and clinical studies suggest that STAT1 also functions as a tumor promoter under specific conditions. In ccRCC, STAT1 activation of JAK2/STAT1/IRF-1 signaling drives the expression of PD-L1 in ccRCC (39). TRAF2 interaction with TNF receptors is required for TNF-alpha-mediated JNK MAP kinase signaling and NF-kappaB activation (40). In addition, TRAF2 regulates inflammatory signaling, thereby affecting the immune response to tumors (41, 42). MAP2K6 is one of the important mitogen-activated protein (MAP) kinase kinases in the MAPK signaling pathway. This protein is involved in cell growth or apoptosis by activating p38 MAP kinase in response to immune stimulation or stress. Our study confirmed the differential expression of MAP2K6 in KIRC, which suggests its possible involvement in the biological processes of ccRCC. Recent study confirms MAP2K6 as senescence-related genes in ccRCC may influence the efficacy of anti-PD-1 therapy and Sunitinib/Everolimus treatment (43). Related studies have confirmed that activation of the Ras-MAPK pathway promotes immune evasion of tumor cells, proving that many associated molecules of the MAPK signaling pathway are significantly correlated with immune cell infiltration. MAPK pathway-targeting inhibitors combined with immune drugs can enhance anti-tumor immunity (44). Meanwhile, this study confirmed the alteration of multiple immune cell infiltrations including CD4_T, CD4_naive, and CD8_naive in the immune microenvironment of KIRC. The above studies on the regulation of MAPK signaling-related genes in different tumors for inflammatory cell infiltration and for PD-1/PD-L1 expression seem to explain the changes in immune cell infiltration in ccRCC.

We divided KIRC patients into high-risk and low-risk groups based on this risk model, and KIRC patients in the high-risk group had a lower survival rate than KIRC patients in the low-risk group. The ROC curve calculation results proved the high accuracy of the risk model. We validated the relationship between the risk model and the clinical characteristics of the patients and the results suggest that prognostic model genes influence the tumor volume (T), lymph node (N) distant metastasis (M) of KIRC patients. After identifying age, stage, grading and risk score as the four independent risk factors for KIRC, we drew a nomogram based on these independent risk factors. We could judge the 5-, 7- or 10-year survival of the KIRC patients based on this new nomogram. Numerous studies have investigated the role of MAPK pathway-related genes in different cancers.

In summary, the pathogenesis of KIRC and various cancers are related to the signal changes of the MAPK signaling pathway. The development of drugs acting on this pathway may provide new ideas for treating KIRC and cancer. Research in this field has confirmed that abnormal activation of MAPK is related to tumor cell invasion, migration, proliferation, apoptosis and degradation of extracellular matrix (45). A deeper understanding of the mechanism of action of the MAPK pathway on cancer, especially KIRC, may become the direction of future basic research.

## 5 Conclusions

In our research, we used 14 genes related to the MAPK signaling pathway to establish a new KIRC predictive risk model, and the role of the ROC curve is to predict the accuracy of the model (5-year AUC value of 0.744, 10-year AUC value of 0.825), suggesting that the model has good predictive performance. However, it must be acknowledged that the specific mechanism of how these 14 genes function in KIRC is not yet clear. In addition, this prognostic risk model needs to be further validated using large-scale multi-center clinical data. However, we firmly believe our study can provide valuable consultation for future scientific diagnosis and clinical treatment of KIRC.

## Data availability statement

The original contributions presented in the study are included in the article/supplementary material. Further inquiries can be directed to the corresponding author.

## Author contributions

QX and GW designed the research methods. PZ and JL participated in data collection and analyzed the data. ZW, LZ and PZ drafted the manuscript. YX and JL revised the manuscript. PZ and JQ participated in the execution of the experiments. All authors contributed to the article and approved the submitted version.

## Glossary

**Table:**

RTKs	receptor tyrosine kinases
GEFs	GTP/GDP exchange factors
CTRP	The Cancer Therapeutics Response Portal
GTEx	Genotype-Tissue Expression
PPI	Protein-protein interaction
ERK	extracellular regulated protein kinases
TGFB1	transforming growth factor-beta 1
MKNK1	MAPK interacting serine/threonine kinase 1
MAP3K10	mitogen-activated protein kinase kinase kinase 10
STAT1	signal transducer and activator of transcription 1
MAP3K8	mitogen-activated protein kinase kinase kinase 8
RPS6KB2	ribosomal protein S6 kinase B2
SHC1	SHC adaptor protein 1
TGFB3	transforming growth factor-beta 3
MAP3K9	mitogen-activated protein kinase kinase kinase 9
MAP4K1	mitogen-activated protein kinase kinase kinase kinase 1
TRAF2	TNF receptor associated factor 2
RAC1	Rac family small GTPase 1)
MAP3K12	mitogen-activated protein kinase kinase kinase 12
IKBKB	inhibitor of nuclear factor kappa B kinase subunit beta
MAP2K2	mitogen-activated protein kinase kinase 2
RPS6KA4	ribosomal protein S6 kinase A4
MAPK3	mitogen-activated protein kinase 3
MAPK9	mitogen-activated protein kinase 9
MAP3K1	mitogen-activated protein kinase kinase 1
MAPK8	mitogen-activated protein kinase 8
MAPK1	mitogen-activated protein kinase 1
MAP2K6	mitogen-activated protein kinase kinase 6
MAPK13	mitogen-activated protein kinase 13
MAP3K5	mitogen-activated protein kinase kinase 5
MAP3K13	mitogen-activated protein kinase kinase kinase 13
MAP2K5	mitogen-activated protein kinase kinase 5
CHUK	component of inhibitor of nuclear factor kappa B kinase complex
RAPGEF2	Rap guanine nucleotide exchange factor 2
RPS6KA2	ribosomal protein S6 kinase A2
RPS6KA5	ribosomal protein S6 kinase A5
MAP2K4	mitogen-activated protein kinase kinase 4
MAP4K1	mitogen-activated protein kinase kinase kinase kinase 1
RPS6KA3	ribosomal protein S6 kinase A3
MAPK5	mitogen-activated protein kinase 5
MAPK7	mitogen-activated protein kinase 7
MAPK10	mitogen-activated protein kinase 10
ELK1	ETS transcription factor ELK1
CREB1	cAMP responsive element binding protein 1
NFKBIA	NFKB inhibitor alpha
ATF2	activating transcription factor 2
NFKB1	nuclear factor kappa B subunit 1
ACC	Adrenocortical carcinoma
BRCA	Breast invasive carcinoma
BLCA	Bladder Urothelial Carcinoma
KICH	Kidney Chromophobe
KIRP	Kidney renal papillary cell carcinoma
