# Supplementary material for: Establishment of a new prognostic risk model of MAPK pathway-related molecules in kidney renal clear cell carcinoma based on genomes and transcriptomes analysis
Source: Front Oncol. 2023 Mar 10;13:1077309. doi: 10.3389/fonc.2023.1077309 (PMC10036835; doi:10.3389/fonc.2023.1077309)
Supplement: Supplementary file 1 [file DataSheet_1.zip › Raw Data/CNV/f3a3b92e-92a8-4125-8990-634ed963a02c.pdf]

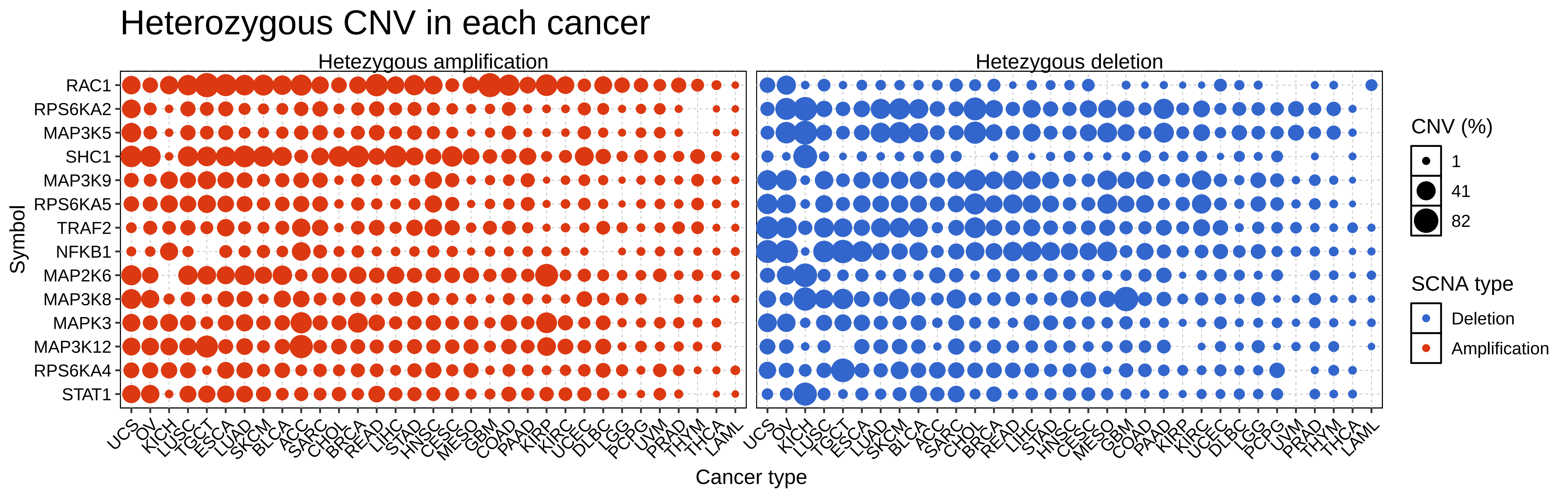

Heterozygous CNV in each cancer
Hetezygous amplification
Hetezygous deletion
RAC1
RPS6KA2
MAP3K5
SHC1
MAP3K9
RPS6KA5
TRAF2
NFKB1
MAP2K6
MAP3K8
MAPK3
MAP3K12
RPS6KA4
STAT1
Symbol
UCS
OV
KICH
LUSC
TGCT
ESCA
LUAD
SKCM
BLCA
ACC
SARC
CHOL
BRCA
READ
LIHC
STAD
HNSC
CESC
MESO
GBM
COAD
PAAD
KIRP
KIRC
UCEC
DLBC
LGG
PCPG
UVM
PRAD
THYM
THCA
LAML
Cancer type
CNV (%)
1
41
82
SCNA type
Deletion
Amplification
