# Supplementary material for: Establishment of a new prognostic risk model of MAPK pathway-related molecules in kidney renal clear cell carcinoma based on genomes and transcriptomes analysis
Source: Front Oncol. 2023 Mar 10;13:1077309. doi: 10.3389/fonc.2023.1077309 (PMC10036835; doi:10.3389/fonc.2023.1077309)
Supplement: Supplementary file 1 [file DataSheet_1.zip › Raw Data/SNV/c754adec-2ed2-404c-828d-16e3bfb7bbce.pdf]

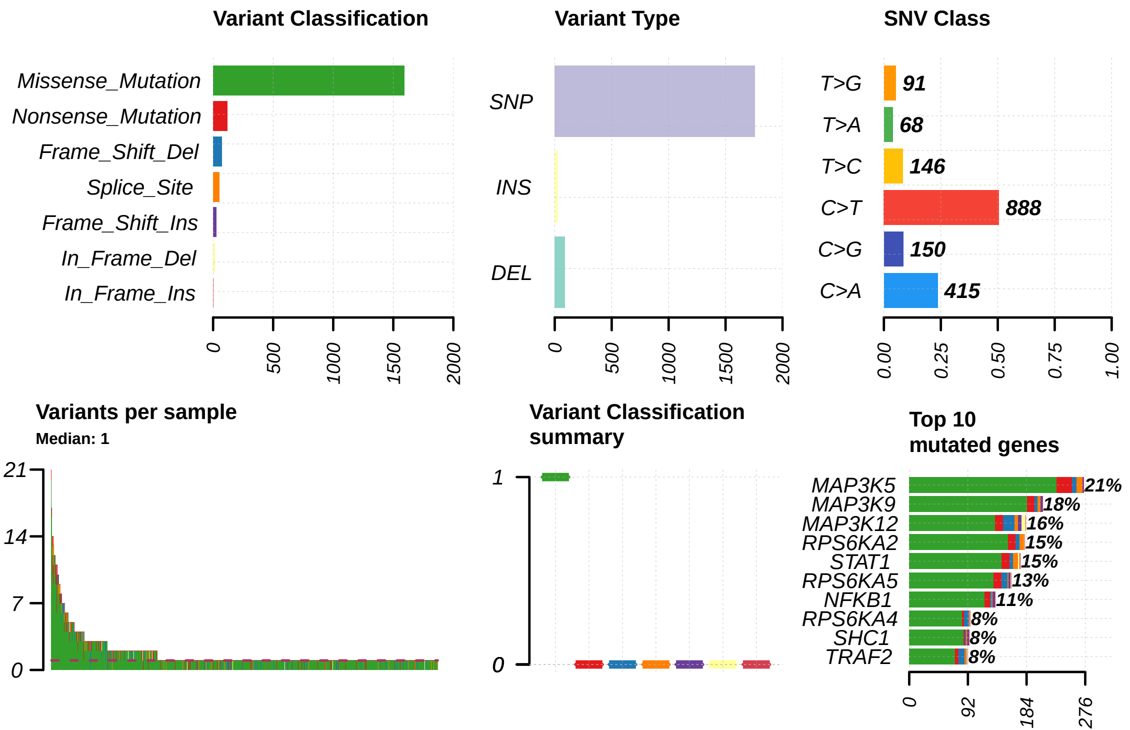
Variant Classification
Missense_Mutation
Nonsense_Mutation
Frame_Shift_Del
Splice_Site
Frame_Shift_Ins
In_Frame_Del
In_Frame_Ins
0
500
1000
1500
2000
Variant Type
SNP
INS
DEL
0
500
1000
1500
2000
SNV Class
T>G 91
T>A 68
T>C 146
C>T 888
C>G 150
C>A 415
0.00
0.25
0.50
0.75
1.00
Variants per sample
Median: 1
21
14
7
0
Variant Classification summary
1
0
Top 10 mutated genes
MAP3K5 21%
MAP3K9 18%
MAP3K12 16%
RPS6KA2 15%
STAT1 15%
RPS6KA5 13%
NFKB1 11%
RPS6KA4 8%
SHC1 8%
TRAF2 8%
0
92
184
276
