# Supplementary figures and images for: Establishment of a new prognostic risk model of MAPK pathway-related molecules in kidney renal clear cell carcinoma based on genomes and transcriptomes analysis
Source: Front Oncol. 2023 Mar 10;13:1077309. doi: 10.3389/fonc.2023.1077309 (PMC10036835; doi:10.3389/fonc.2023.1077309)

# CNV percentage in each cancer

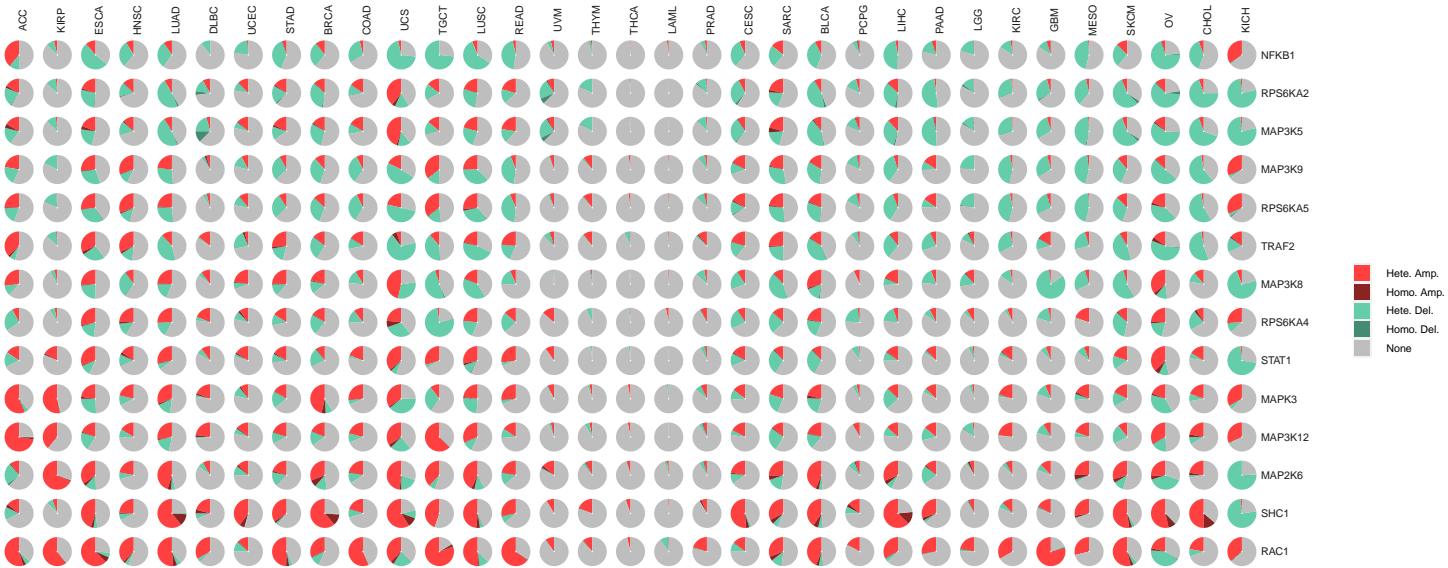

Supplement: Supplementary file 1 [file DataSheet_1.zip › Raw Data/CNV/b9e85630-f7c5-44d8-b26e-d7e4a575bceb.pdf]

# Correlation between CTRP drug sensitivity and mRNA expression

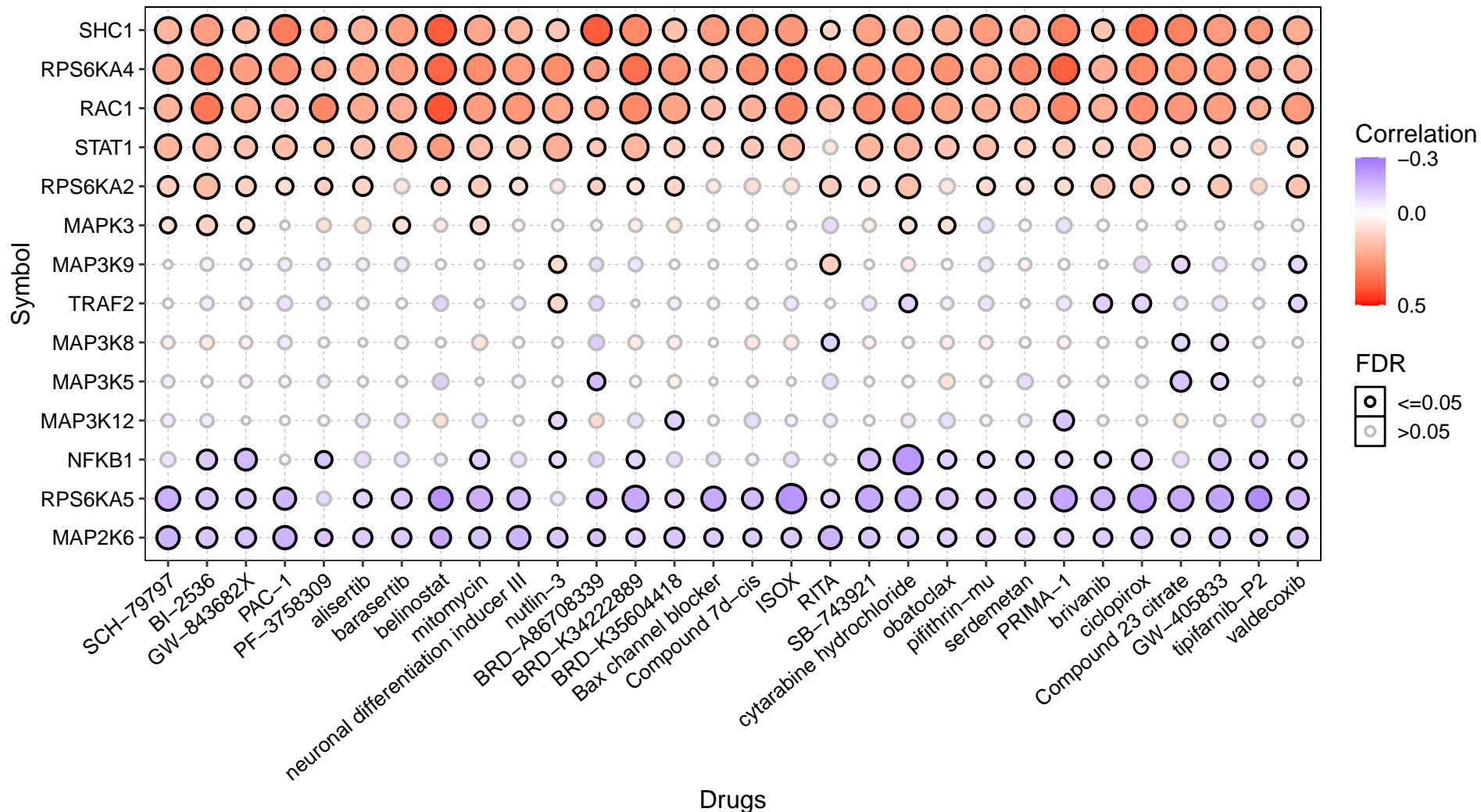

Supplement: Supplementary file 1 [file DataSheet_1.zip › Raw Data/CTRP/2496e7df-6706-4d9b-acf8-82889f4a0f2e.pdf]

Correlation between GDSC drug sensitivity and mRNA expression

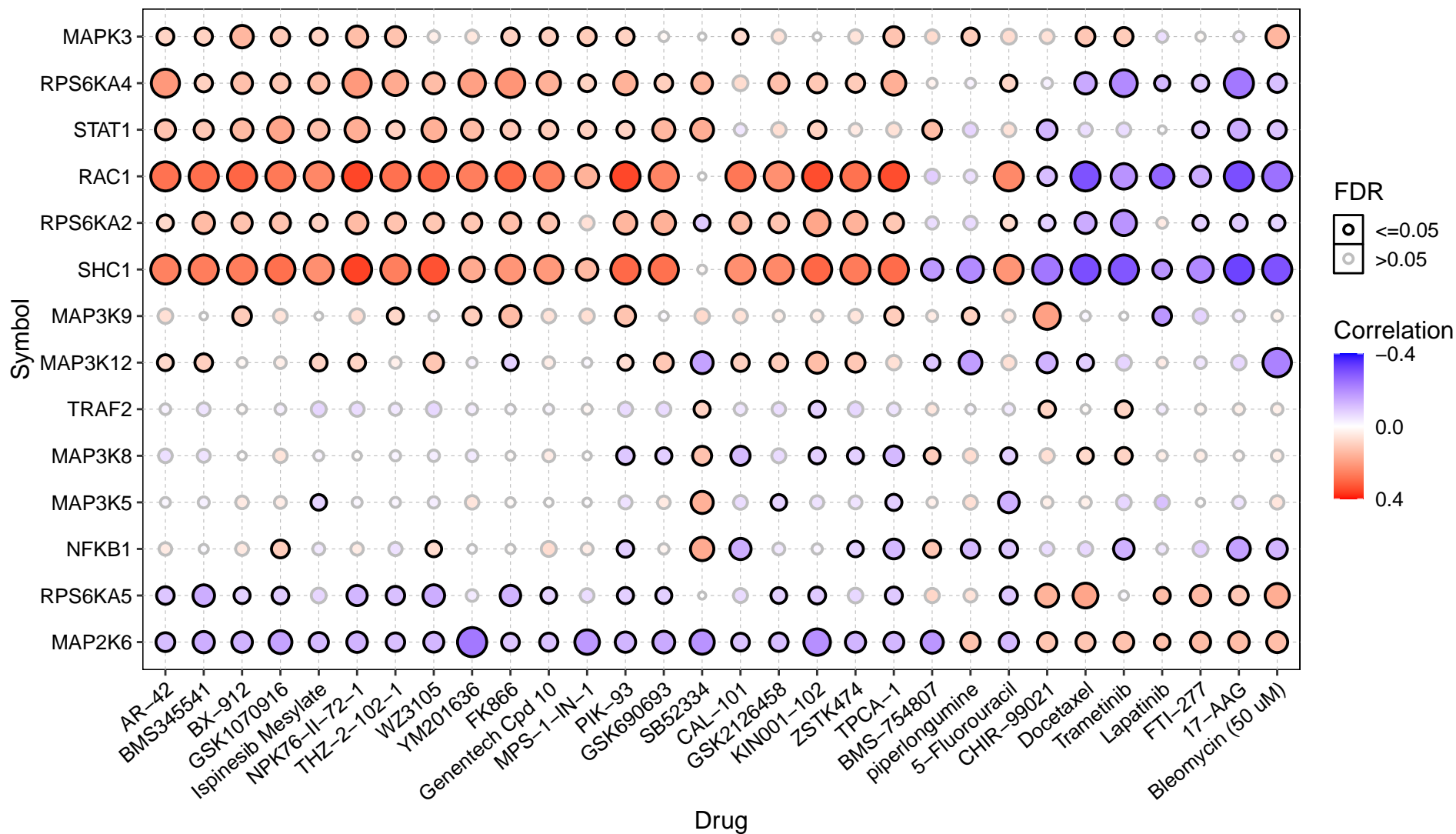

Supplement: Supplementary file 1 [file DataSheet_1.zip › Raw Data/GDSC/312f8068-ddca-42f3-bcc1-113f53fe75bf.pdf]

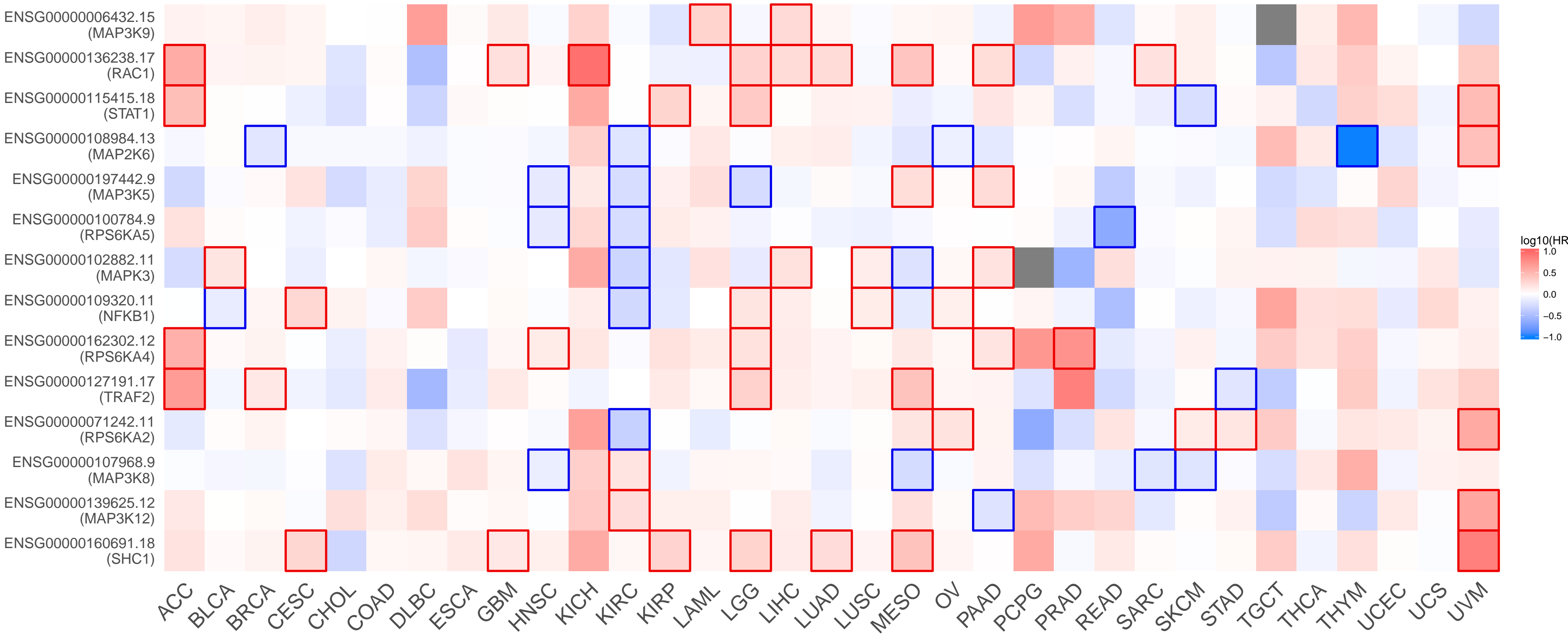

Supplement: Supplementary file 1 [file DataSheet_1.zip › Raw Data/GEPIA/survival_map_eCHQZ.pdf]

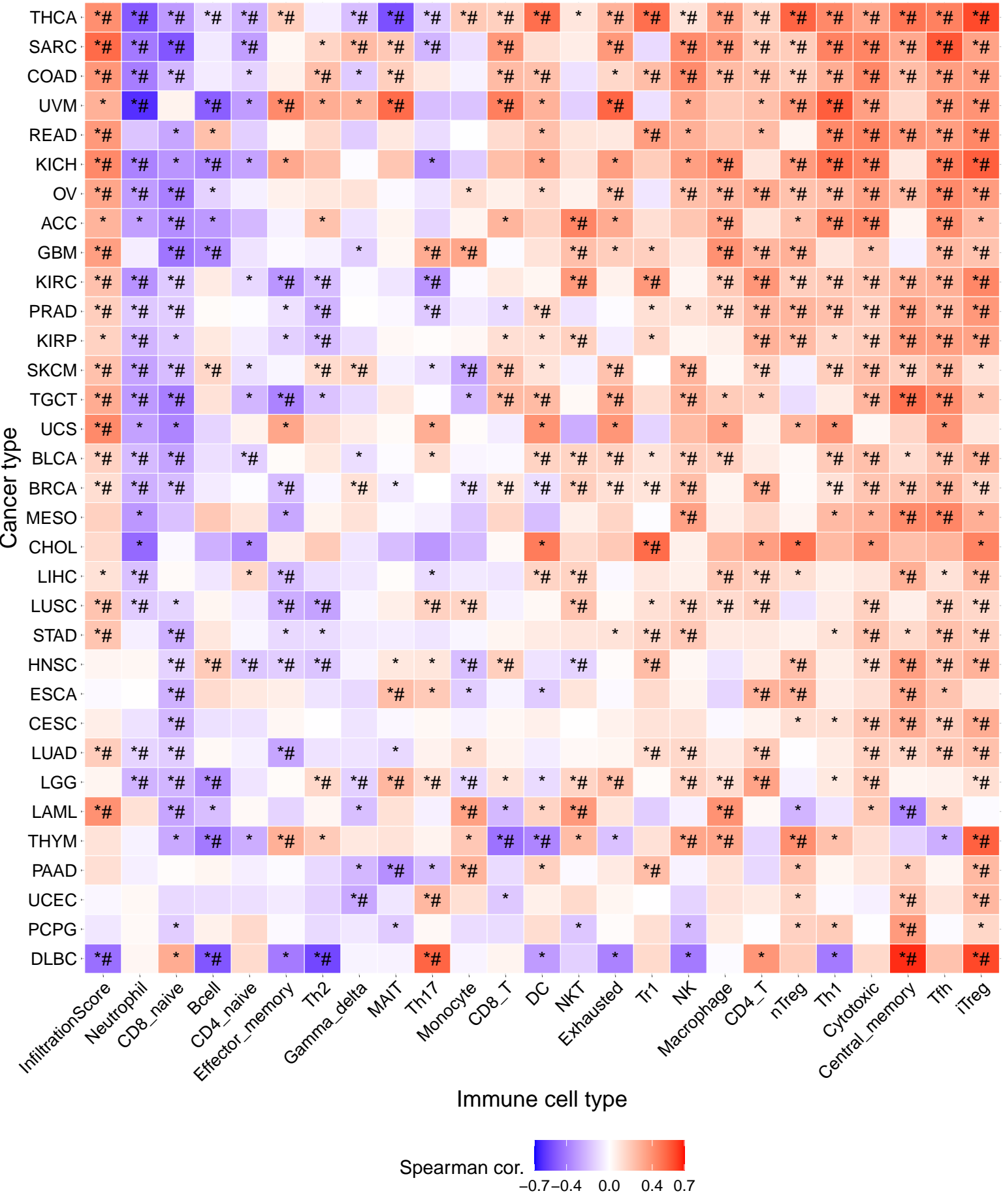

Supplement: Supplementary file 1 [file DataSheet_1.zip › Raw Data/Immunity/d9942f77-e455-43b5-970d-2cf62acd384e.pdf]

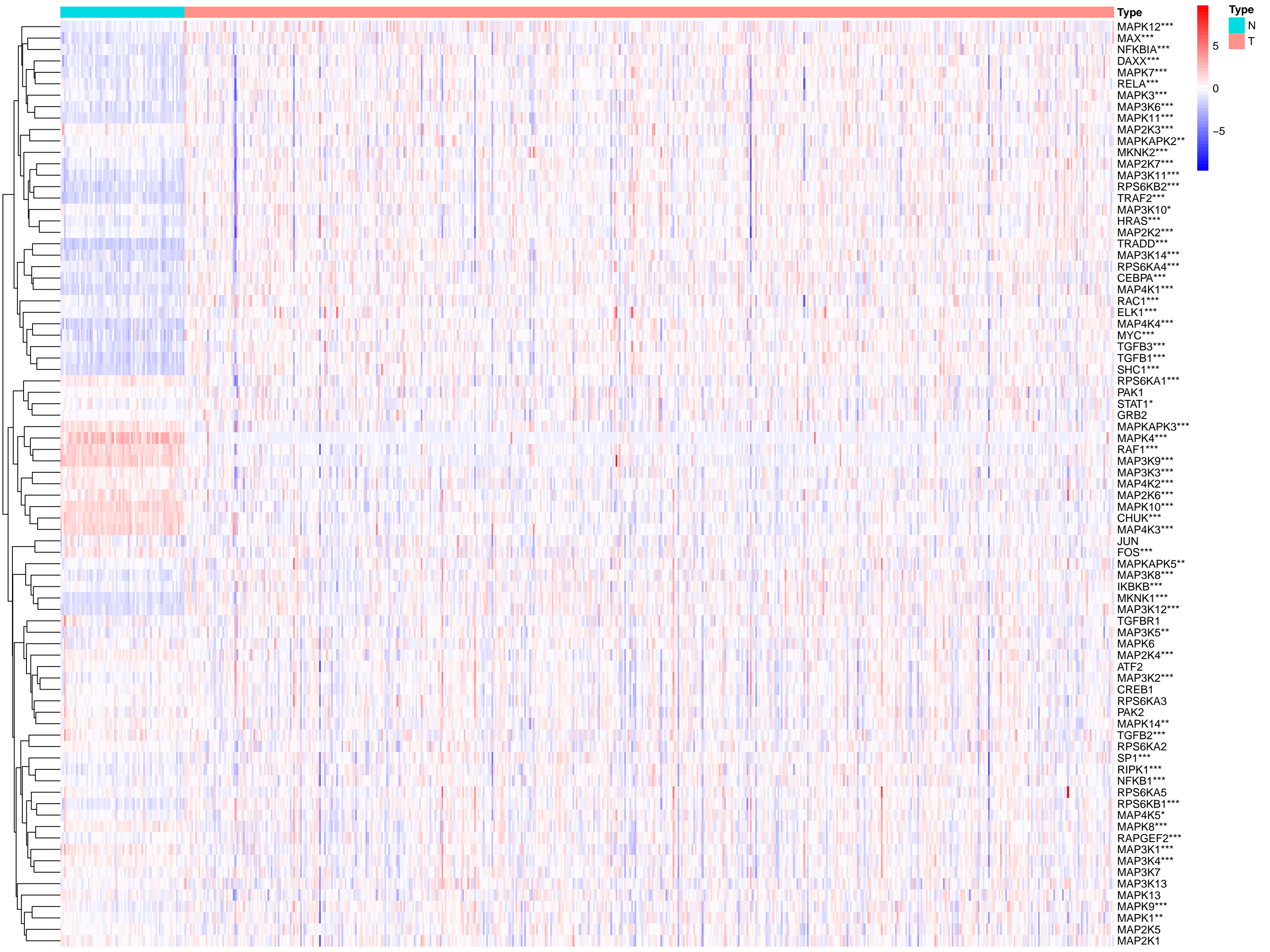

Supplement: Supplementary file 1 [file DataSheet_1.zip › Raw Data/MAPK-KIRC/02.sigPeatmap/heatmap.pdf]

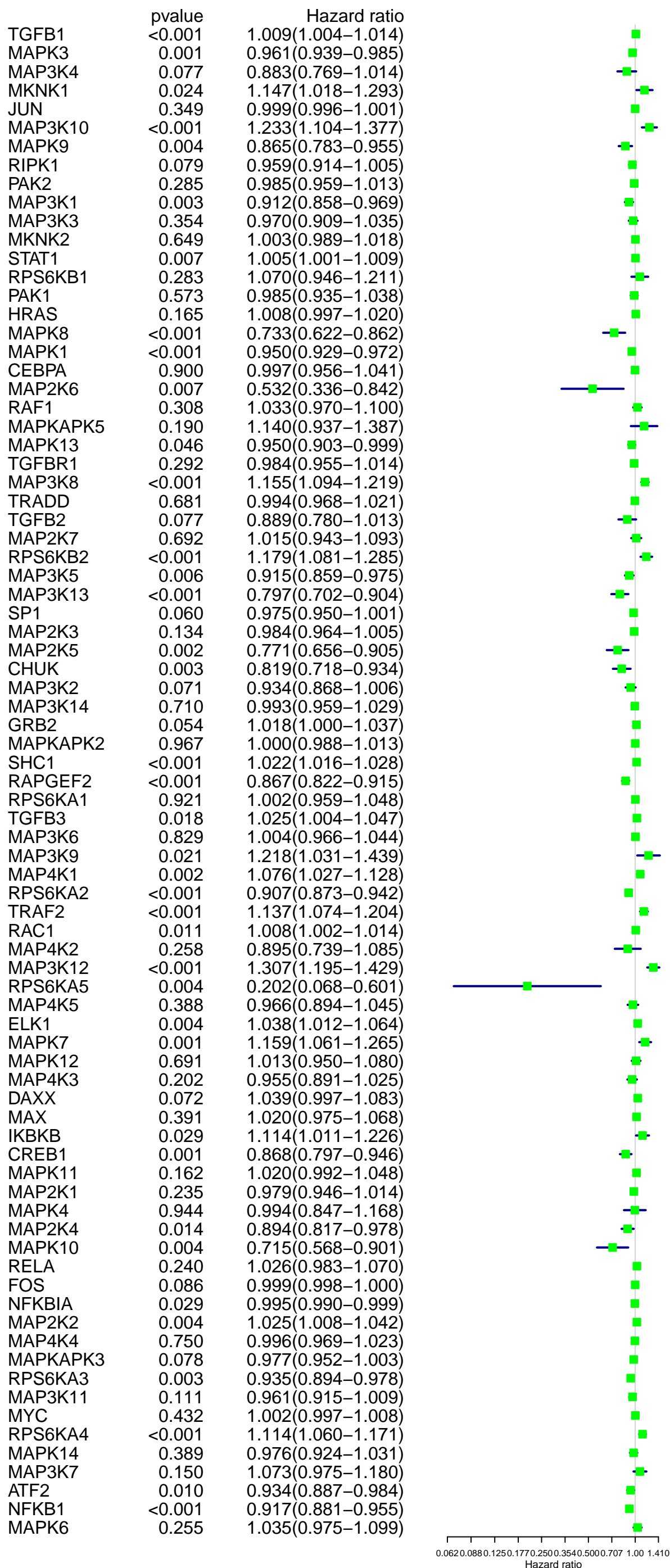

Supplement: Supplementary file 1 [file DataSheet_1.zip › Raw Data/MAPK-KIRC/04.uniCox/forest.pdf]

Partial Likelihood Deviance

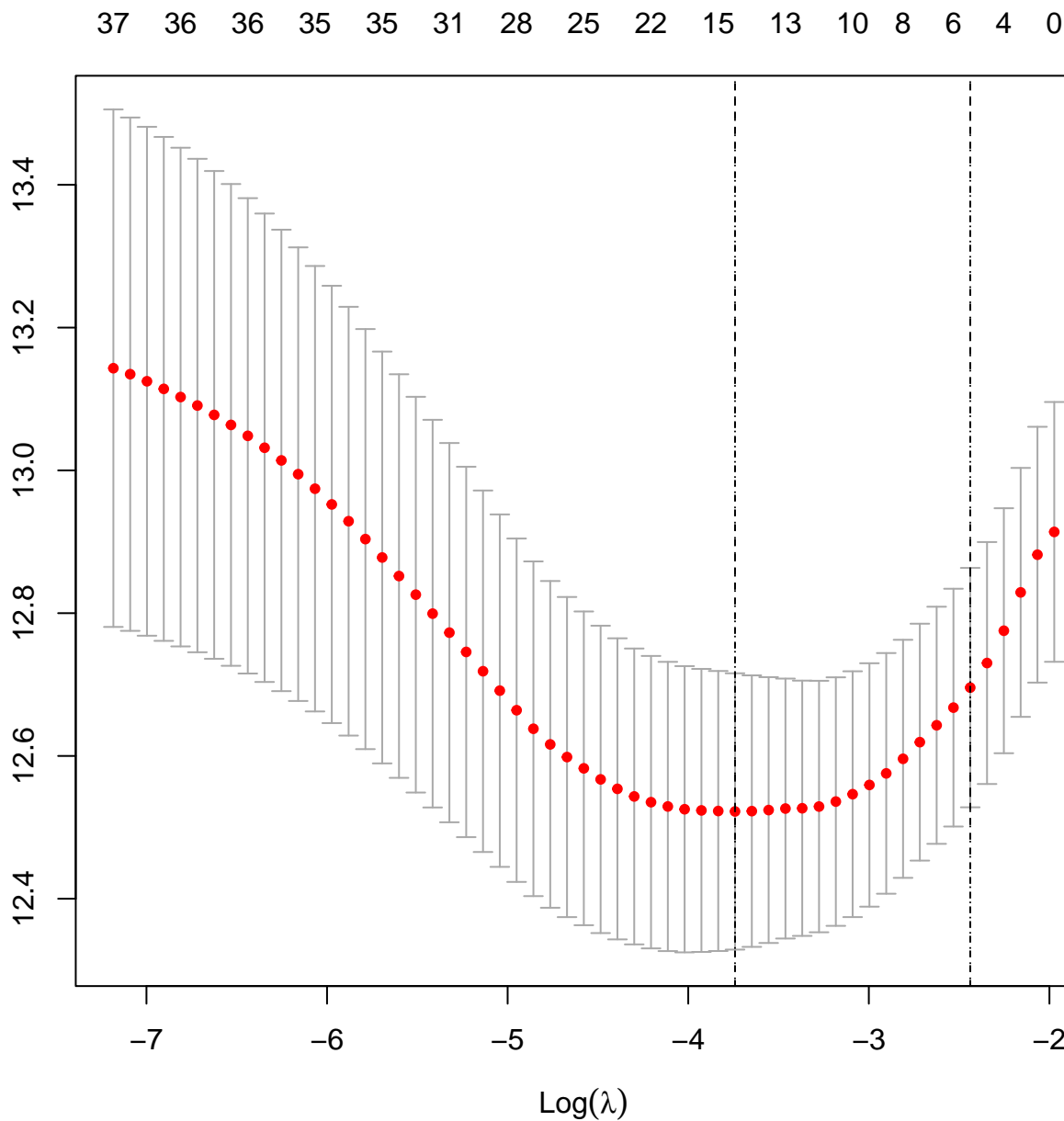

Supplement: Supplementary file 1 [file DataSheet_1.zip › Raw Data/MAPK-KIRC/05.lasso/cvfit.pdf]

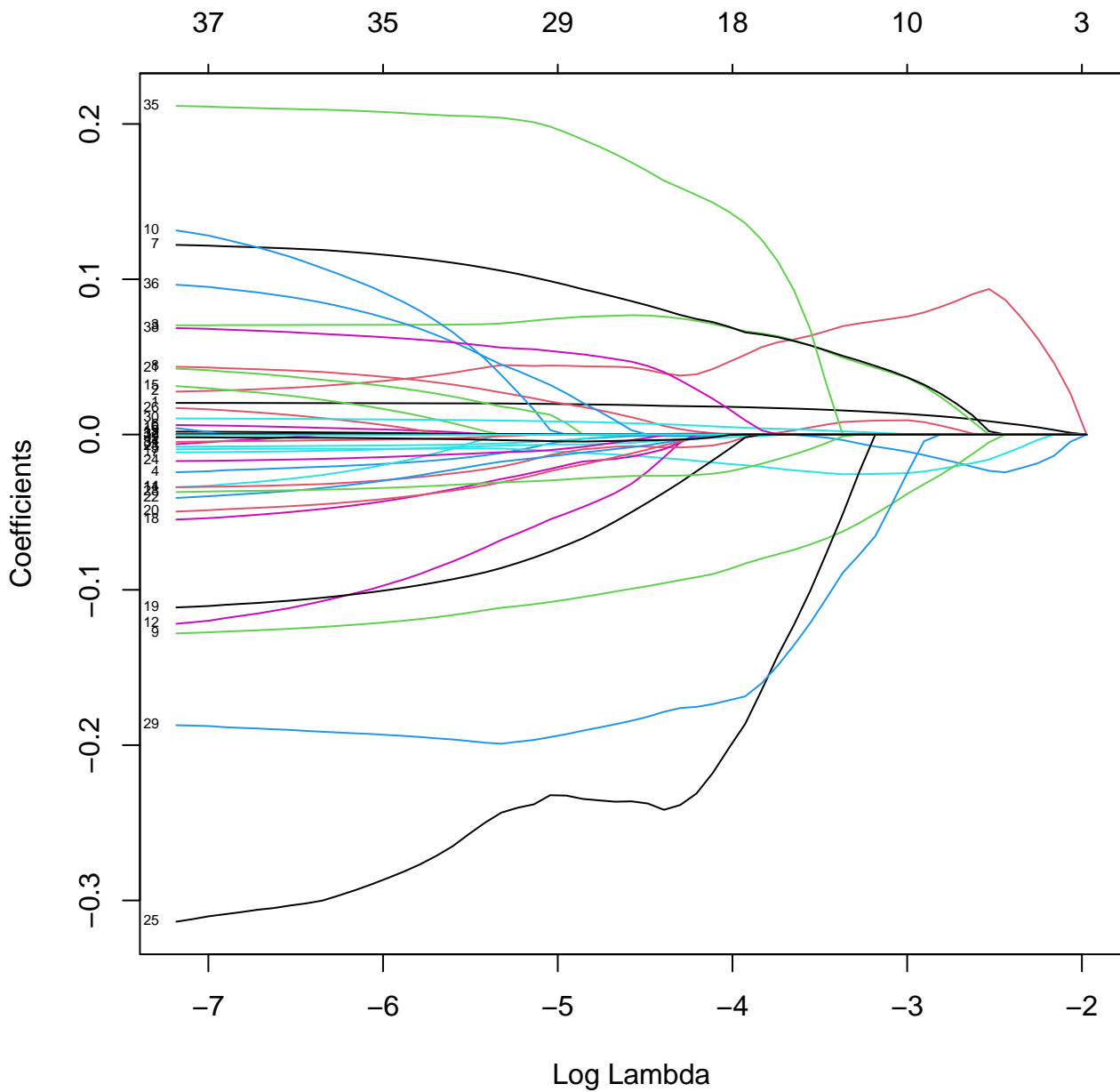

Supplement: Supplementary file 1 [file DataSheet_1.zip › Raw Data/MAPK-KIRC/05.lasso/lambda.pdf]

**Survival curve ( $p=1.847\text{e-}11$ )**

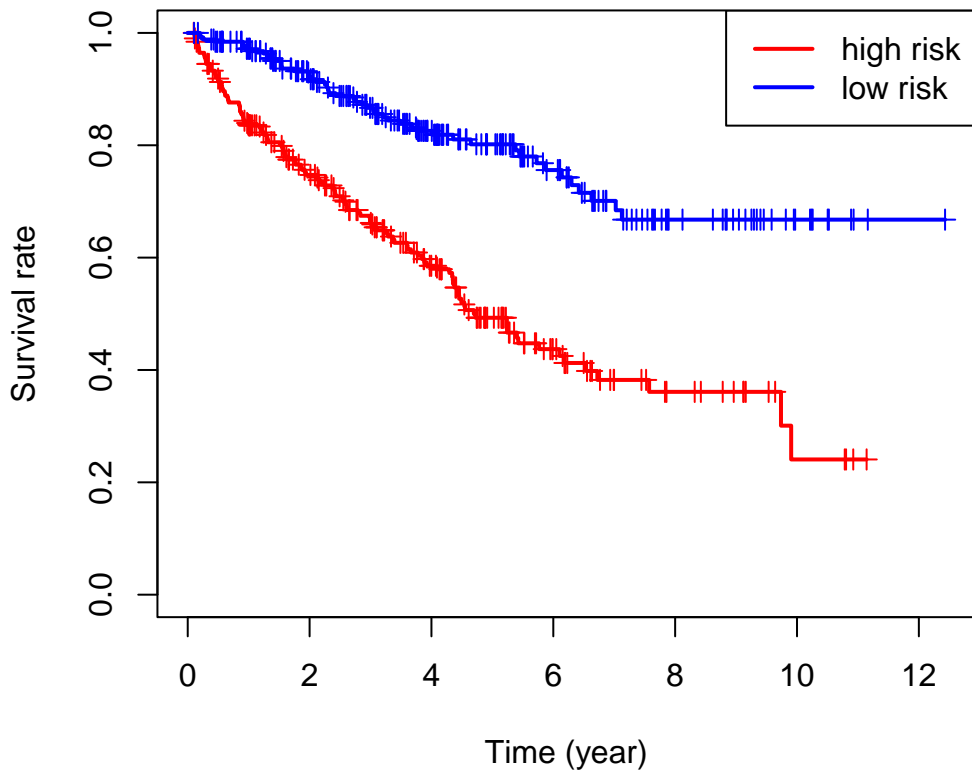

Supplement: Supplementary file 1 [file DataSheet_1.zip › Raw Data/MAPK-KIRC/06.survival/survival.pdf]

**ROC curve ( AUC = 0.825 )**

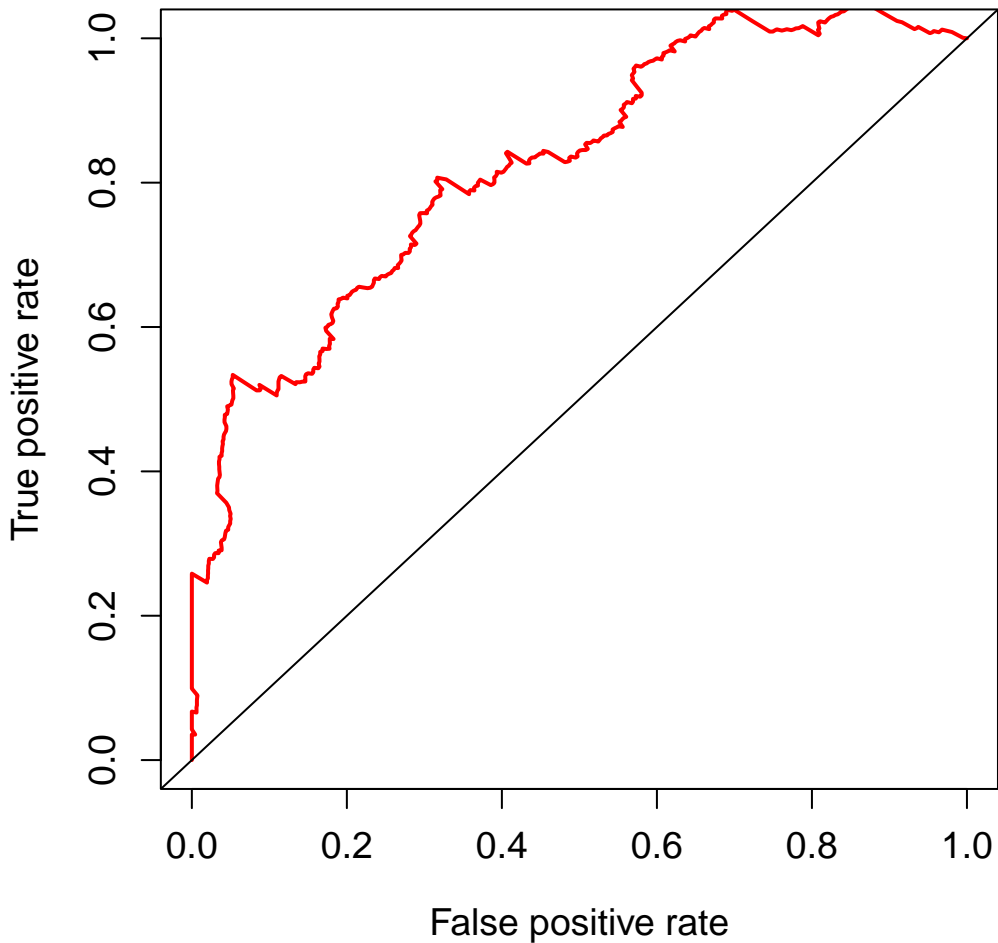

Supplement: Supplementary file 1 [file DataSheet_1.zip › Raw Data/MAPK-KIRC/07.ROC/ROC10.pdf]

**ROC curve ( AUC = 0.744 )**

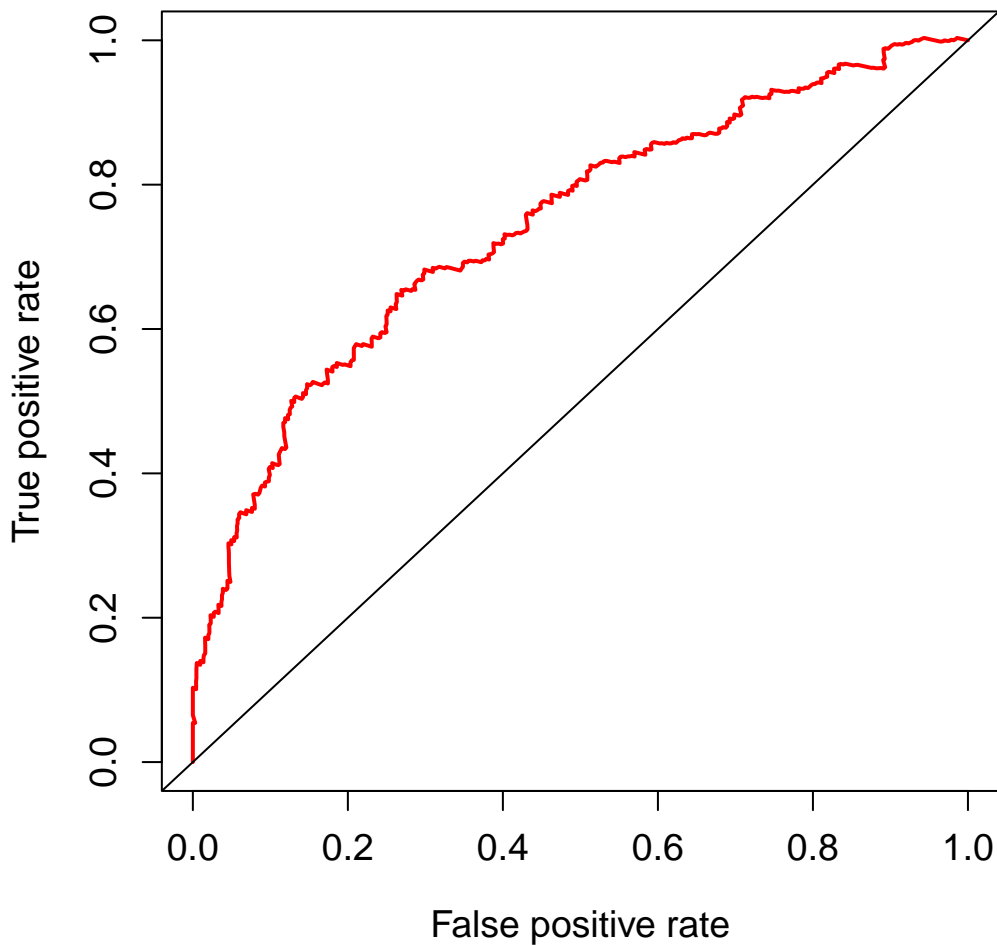

Supplement: Supplementary file 1 [file DataSheet_1.zip › Raw Data/MAPK-KIRC/07.ROC/ROC5.pdf]

**ROC curve ( AUC = 0.744 )**

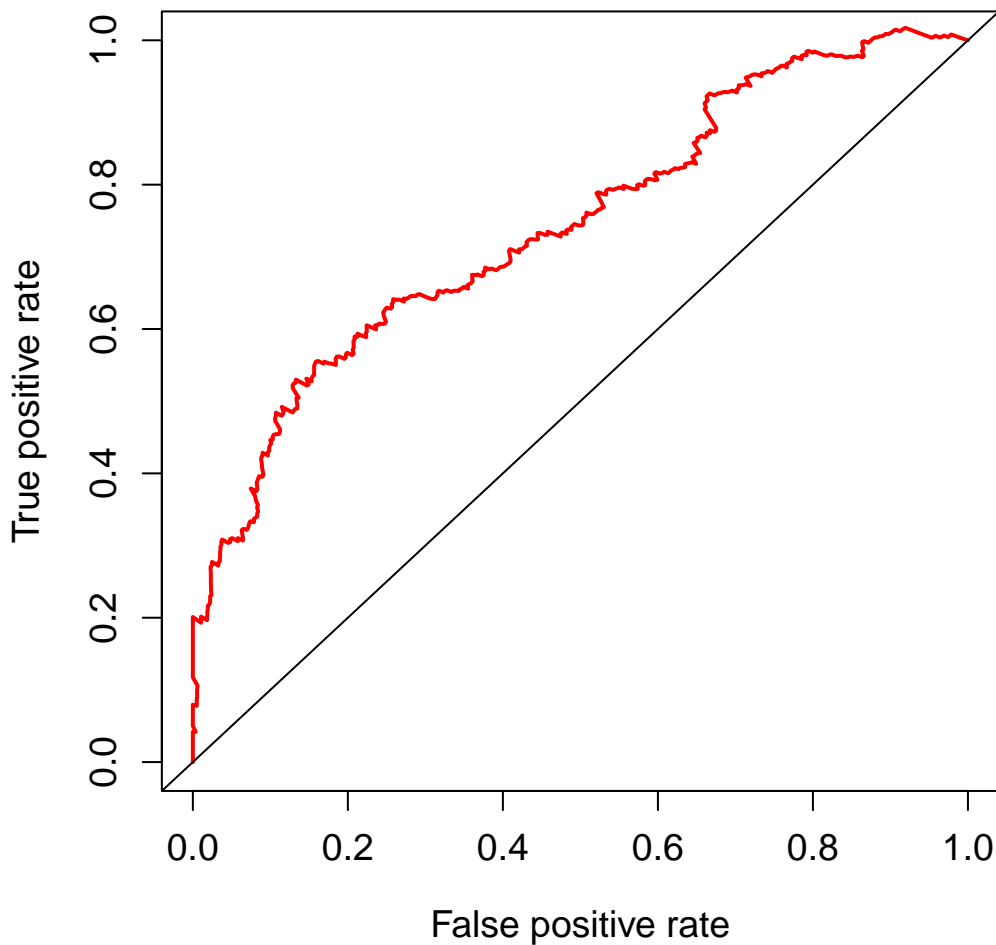

Supplement: Supplementary file 1 [file DataSheet_1.zip › Raw Data/MAPK-KIRC/07.ROC/ROC7.pdf]

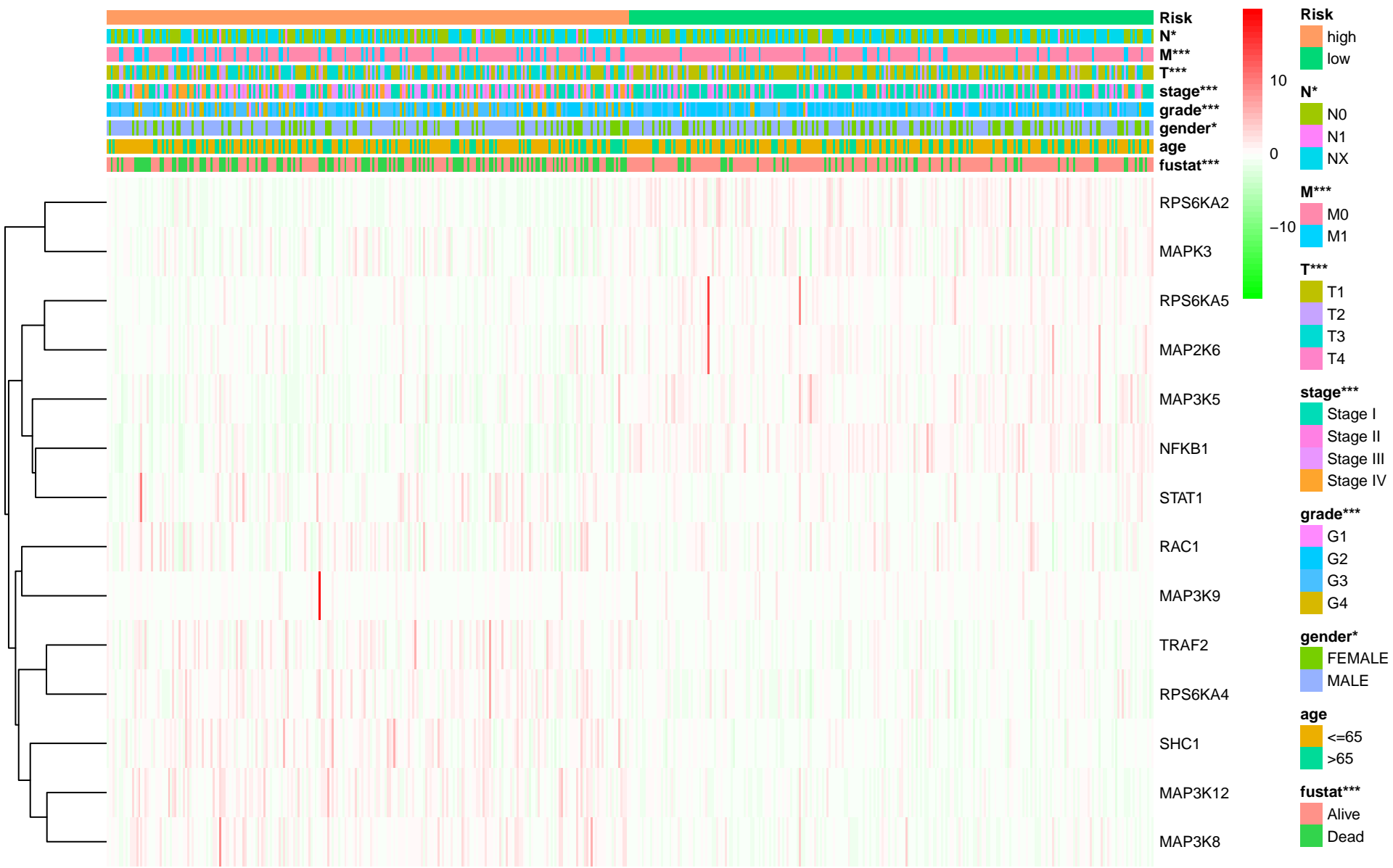

Supplement: Supplementary file 1 [file DataSheet_1.zip › Raw Data/MAPK-KIRC/10.RiskClinicalHeatmap/heatmap.pdf]

|           | pvalue | Hazard ratio       |
|-----------|--------|--------------------|
| age       | <0.001 | 1.028(1.015–1.042) |
| gender    | 0.809  | 0.961(0.697–1.325) |
| grade     | <0.001 | 2.285(1.854–2.816) |
| stage     | <0.001 | 1.900(1.658–2.177) |
| T         | <0.001 | 1.920(1.621–2.273) |
| M         | <0.001 | 4.618(3.366–6.336) |
| riskScore | <0.001 | 1.384(1.302–1.472) |

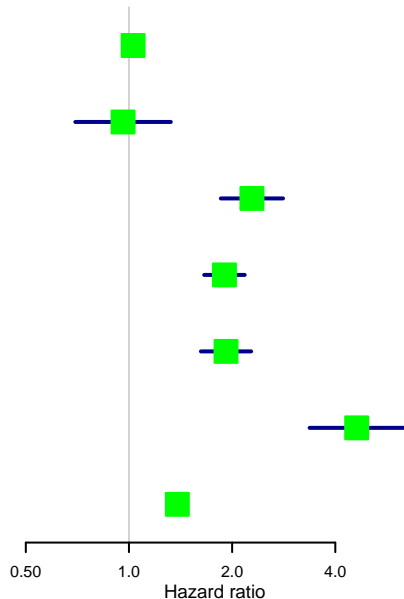

Supplement: Supplementary file 1 [file DataSheet_1.zip › Raw Data/MAPK-KIRC/12.uniIndep/forest.pdf]

|           | pvalue | Hazard ratio       |
|-----------|--------|--------------------|
| age       | <0.001 | 1.031(1.016–1.046) |
| gender    | 0.634  | 1.086(0.773–1.525) |
| grade     | 0.019  | 1.333(1.048–1.697) |
| stage     | 0.048  | 1.603(1.005–2.557) |
| T         | 0.427  | 0.841(0.548–1.291) |
| M         | 0.226  | 1.530(0.768–3.045) |
| riskScore | <0.001 | 1.239(1.156–1.328) |

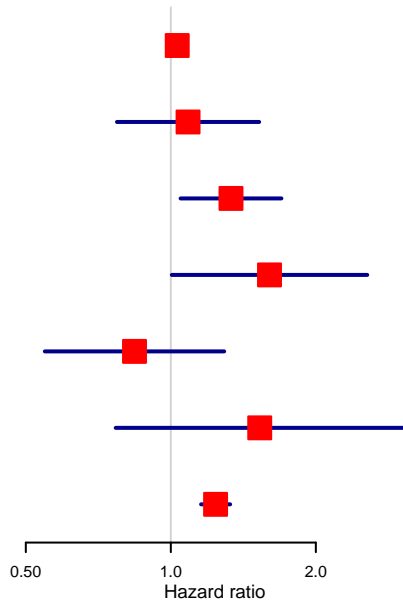

Supplement: Supplementary file 1 [file DataSheet_1.zip › Raw Data/MAPK-KIRC/13.multiIndep/forest.pdf]

Points

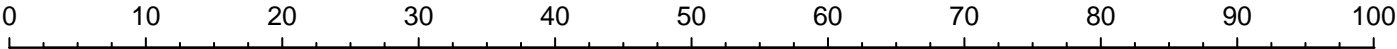

age

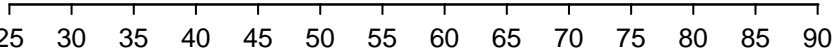

grade

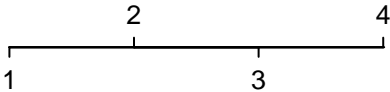

stage

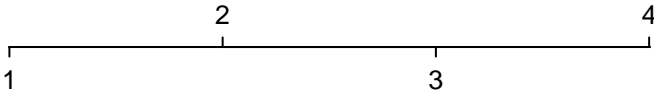

riskScore

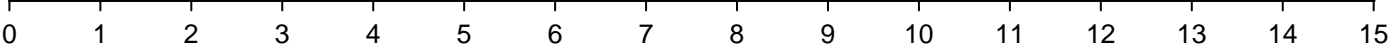

Total Points

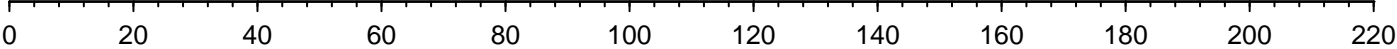

5-year survival

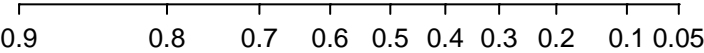

7-year survival

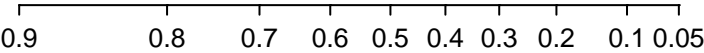

10-year survival

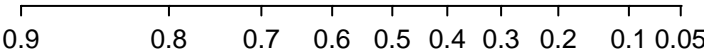

Supplement: Supplementary file 1 [file DataSheet_1.zip › Raw Data/MAPK-KIRC/14.nomogram/tcga.Nomogram.pdf]

# Methylation difference in each cancer

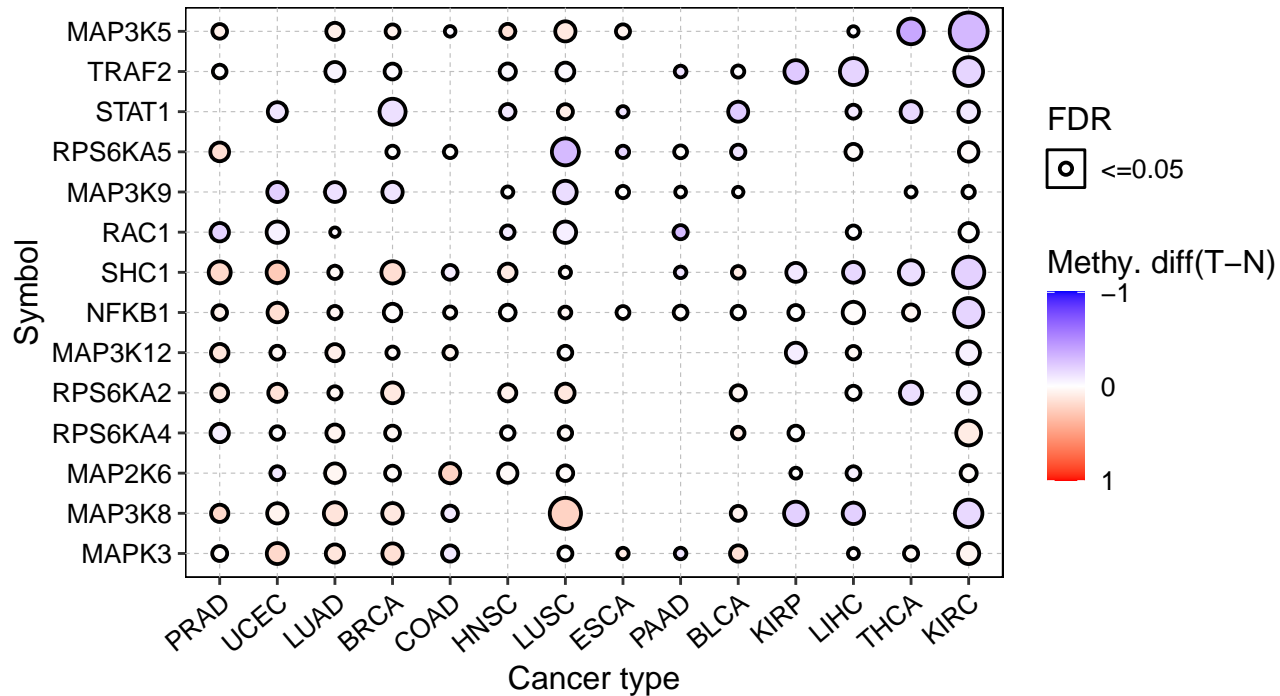

Supplement: Supplementary file 1 [file DataSheet_1.zip › Raw Data/Methylation/c23fdd00-96be-4cf9-9196-6ebc80cb1500.pdf]

SNV percentage heatmap

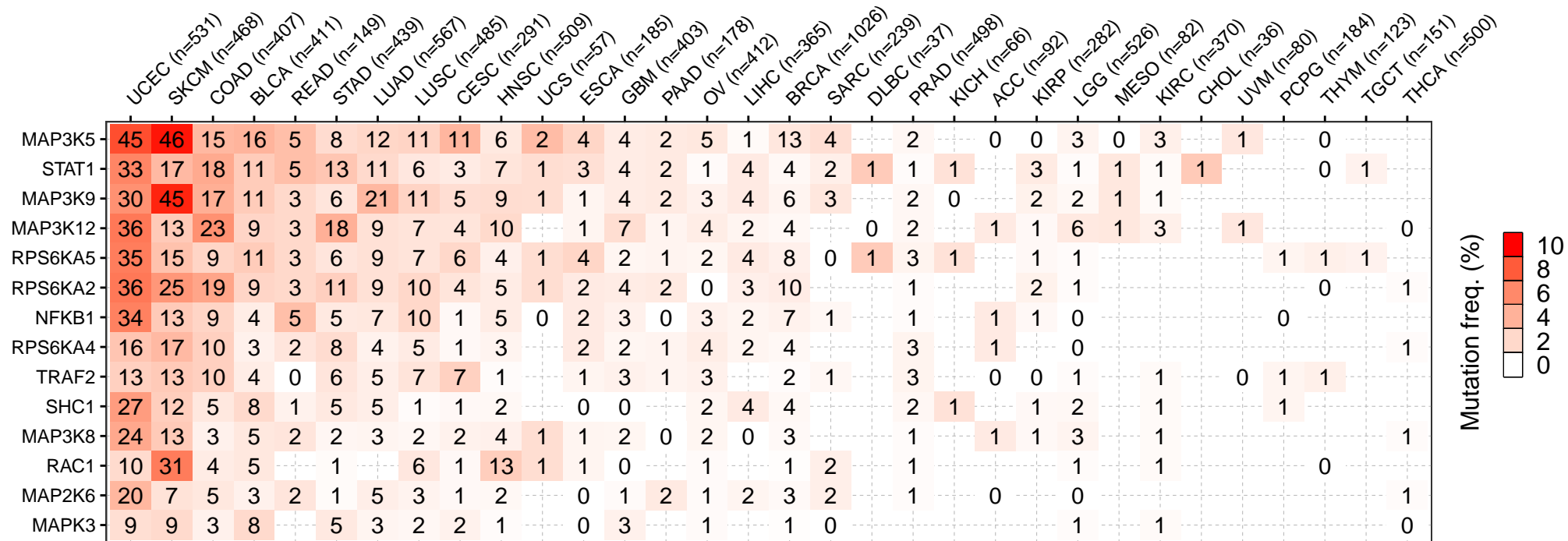

Supplement: Supplementary file 1 [file DataSheet_1.zip › Raw Data/SNV/28ef9314-97ad-4d24-a597-14954079fee8.pdf]

Altered in 934 (87.95%) of 1062 samples.

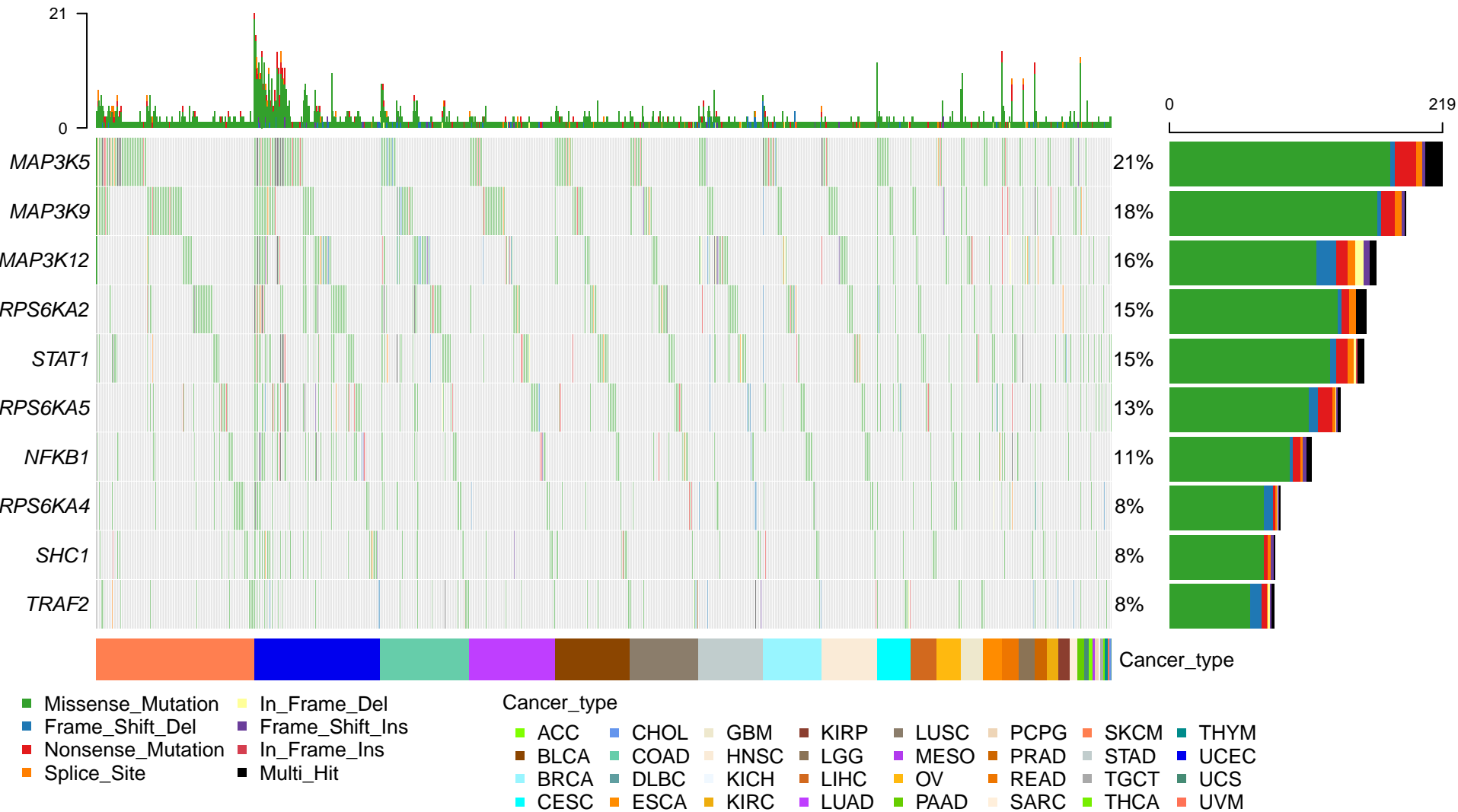

Supplement: Supplementary file 1 [file DataSheet_1.zip › Raw Data/SNV/67da549d-3795-43e6-a258-bc120ba5cc8c.pdf]

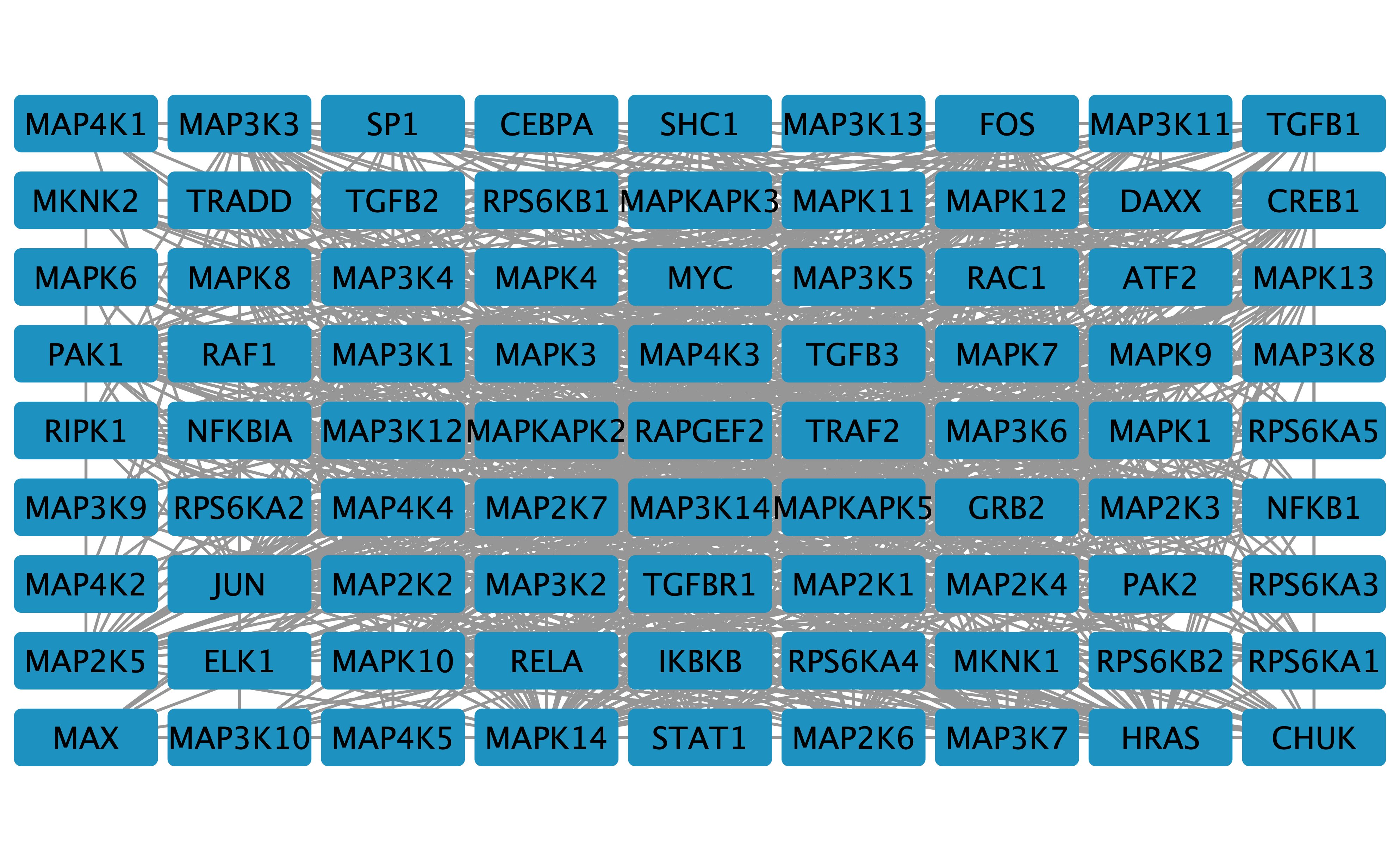

Supplement: Supplementary file 1 [file DataSheet_1.zip › Raw Data/STRING/MAPK.png]

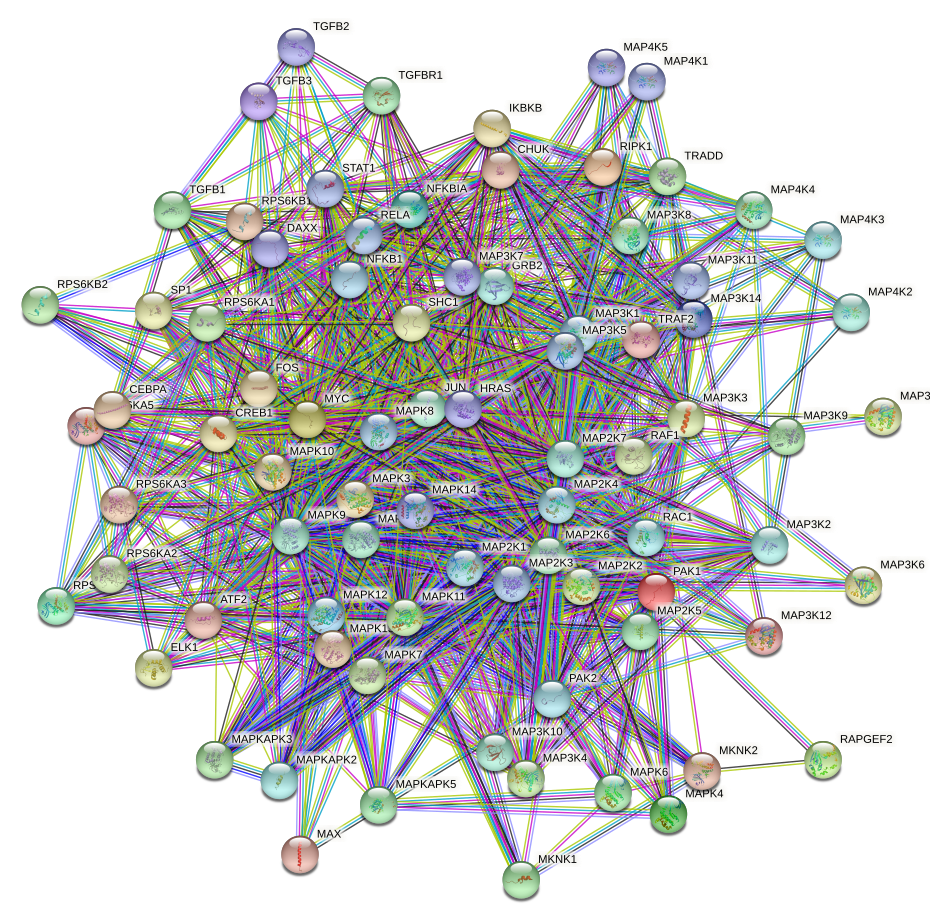

Supplement: Supplementary file 1 [file DataSheet_1.zip › Raw Data/STRING/string_normal_image.png]
