# Supplementary material for: Establishment of a new prognostic risk model of MAPK pathway-related molecules in kidney renal clear cell carcinoma based on genomes and transcriptomes analysis
Source: Front Oncol. 2023 Mar 10;13:1077309. doi: 10.3389/fonc.2023.1077309 (PMC10036835; doi:10.3389/fonc.2023.1077309)
Supplement: Supplementary file 1 [file d2d1dff5-dd9f-4501-b93d-2129936a829d.pdf › Raw Data/CNV/d2d1dff5-dd9f-4501-b93d-2129936a829d.pdf]

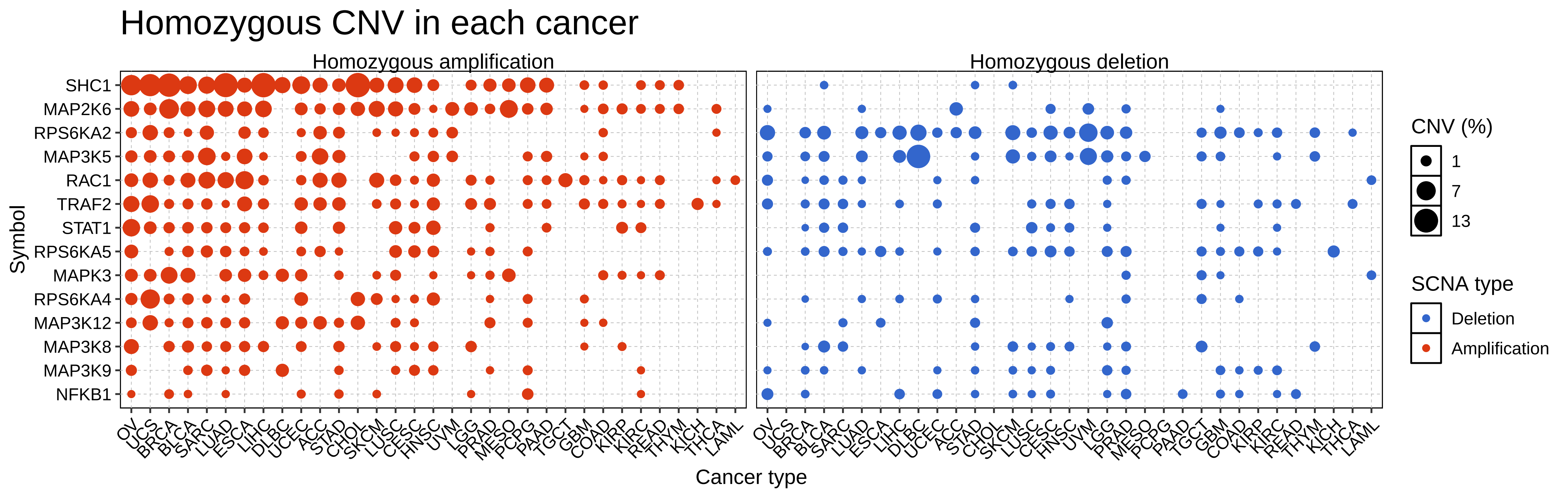

Homozygous CNV in each cancer
Homozygous amplification
Homozygous deletion
SHC1
MAP2K6
RPS6KA2
MAP3K5
RAC1
TRAF2
STAT1
RPS6KA5
MAPK3
RPS6KA4
MAP3K12
MAP3K8
MAP3K9
NFKB1
Symbol
OV
UCS
BRCA
BLCA
SARC
LUAD
ESCA
LIHC
DLBC
UCEC
ACC
STAD
CHOL
SKCM
LUSC
CESC
HNSC
UVM
LGG
PRAD
MESO
PCPG
PAAD
TGCT
GBM
COAD
KIRP
KIRC
READ
THYM
KICH
THCA
LAML
Cancer type
CNV (%)
1
7
13
SCNA type
Deletion
Amplification
